# The miR-125a-5p/IRF4 Axis Mediates Sodium Arsenite-Induced M2 Macrophage Polarization

**DOI:** 10.3390/biom15111630

**Published:** 2025-11-20

**Authors:** Yan Yu, Fan Yao, Suyuan Tong, Mingzheng Li, Qilong Liao, Fei Wang, Shuhua Xi

**Affiliations:** 1 Key Laboratory of Environmental Stress and Chronic Disease Control and Prevention, Ministry of Education, China Medical University, Shenyang 110122, China; yy1686527@163.com (Y.Y.);; 2 Department of Environmental Health, School of Public Health, China Medical University, Shenyang 110122, China; 3 Key Laboratory of Environmental Pollution Health Risk Assessment, Ministry of Ecology and Environment, South China Institute of Environmental Sciences, Guangzhou 510655, China; 4 Key Laboratory of Liaoning Province on Toxic and Biological Effects of Arsenic, China Medical University, Shenyang 110122, China; 5 Liaoning Key Laboratory of Environmental Health Damage Research and Assessment, Shenyang 110122, China

**Keywords:** sodium arsenite, macrophage polarization, MiR-125a, IRF4

## Abstract

Arsenic, a ubiquitous metalloid, is commonly found in surface waters; as well as serious human health issues, it also induces systemic diseases and carcinogenesis upon chronic exposure. To better understand how arsenic potentially alters the immune system, it is important to study its effects on macrophage polarization. Micro-RNA plays an epigenetic regulatory role in organisms. The miR-125 family regulates macrophage polarization and tumorigenesis, yet its role in arsenic-induced macrophage polarization remains unexplored. This study investigated the mechanism of sodium arsenite (NaAsO_2_)-driven macrophage polarization via miR-125a-5p. In vivo, rats exposed to 10 or 50 mg/L NaAsO_2_ for 12 weeks exhibited elevated M2 markers (CD206, Arg1) and reduced M1 markers (iNOS, IL-1β, TNF-α) in liver and bladder tissues. In vitro, THP-1-derived macrophages treated with NaAsO_2_ (2–8 μM) for 48 h showed dose-dependent M2 polarization, marked by upregulated CD206, Arg1, and IL-10. Flow cytometry results show that the proportion of M2/M1-type cells has increased significantly. Notably, NaAsO_2_ suppressed miR-125a-5p expression and elevated interferon regulatory factor 4 (IRF4), a predicted target of miR-125a-5p. Overexpression of miR-125a-5p reversed NaAsO_2_-induced M2 polarization by inhibiting IRF4, thereby reducing M2 markers and restoring M1-associated proteins. These findings reveal that NaAsO_2_ promotes M2 macrophage polarization through the miR-125a-5p/IRF4 axis, highlighting a novel epigenetic mechanism in arsenic-associated tumor microenvironments and immune dysfunction. This study provides critical insights into targeting miR-125a-5p as a therapeutic strategy.

## 1. Introduction

Arsenic is generally found in the natural environment in the form of trivalent and pentavalent compounds. The primary way that people are exposed to an environment with high levels of arsenic is by drinking groundwater that has been contaminated by naturally occurring arsenic. The standard level of arsenic in drinking water recommended by the World Health Organization is 10 μg/L. Around the world, a large number of people are affected by high-arsenic groundwater, and China is one of the countries suffering from arsenic pollution [1]. According to epidemiological research, the main harmful effects of prolonged exposure to arsenic include immunotoxicity, neurotoxicity, and toxicity to reproduction and development. It can also cause a number of human cancers, including skin, lung, and bladder cancer [2,3,4]. However, the mechanism is still unclear.

Macrophages are an important component of the innate immune system, which play a vital role in physiological processes such as fighting inflammation, self-defense, and tissue repair. The surrounding environment affects macrophages by changing their phenotype and thus affecting their function, which can be mainly divided into M1 and M2 [5]. M1 macrophages play an important role in the initial stage of inflammation by recognizing invading pathogens and rapidly phagocytosing and eliminating them, activating an inflammatory response to protect the body. M2 macrophages have anti-inflammatory and repair effects on damaged tissues [6]. The cross-talk between the M1 and M2 macrophage polarization processes, mainly based on the balance between the activation of STAT1 and STAT3/STAT6, regulates and plays a role in the polarization classification of macrophages [7].

A large number of studies have shown that miRNAs are related to macrophage polarization [8]. Among them, the miR-125 subfamily exists in macrophages, which can inhibit the transformation of macrophages to M2 and promote the transformation to M1, enhance the destruction of tumor cells, and thus inhibit the development of tumors [9,10]. MiR-125a promotes the pro-inflammatory effect of M1 macrophages through the TNFAIP3/NFκB signaling pathway. MiR-125b can directly act on the 3′-UTR region of TNF-α mRNA to downregulate the expression of TNF-α and regulate the inflammatory response [11]. Intraperitoneal injection of paclitaxel combined with miRNA-125b has been shown to repolarize macrophages into M1 in patients with ovarian cancer. MiR-125b partially activates macrophages and enhances the functional role of macrophages in inducing an immune response by reducing IRF4 levels [12]. In addition, the miRNA-125 family plays an important role in tumor cells, and its role in cancer depends on the cell type, and it plays a carcinogenic or tumor-suppressor role by affecting the different stages of cell differentiation, proliferation, invasion, and apoptosis [13].

However, the role of the miR-125 family in macrophage polarization and the effects of arsenic on the innate immune cell macrophages remain unclear. Our study aims to explore the role of miR-125a-5p in the polarization of macrophages by establishing the polarization model of arsenic stimulation and to further explore the effects of arsenic on the immune cells. Therefore, we hypothesize that arsenic exposure disrupts the polarization balance of macrophages through epigenetic mechanisms; specifically, arsenic may deactivate the inhibition of IRF4 by downregulating the expression of miR-125a-5p, thereby driving macrophage phenotypic polarization to M2. This propensity for M2 polarization may lead to immune dysfunction and may play a key role in arsenic-related tumor microenvironment formation and carcinogenesis.

## 2. Materials and Methods

### 2.1. Animal Experimental Model

A total of 18 healthy Wistar rats of about 120 g, aged 6 weeks, were selected and purchased from the Experimental Animal Center of China Medical University. All experimental protocols were approved by the Ethics Review Committee for Animal Experimentation of the China Medical University (CMU2021720, Shenyang, China) and carried out in accordance with the NIH Guide for the Care and Use of Laboratory Animals. The rats were randomly divided into 3 groups, control group, 10 mg/L arsenic group, and 50 mg/L arsenic group, with 6 rats in each group. The rats were given arsenic-containing distilled water to freely drink and were exposed to the poison for 12 weeks. During the feeding period, the hair, appetite, and mental state of the rats were observed daily; the water intake of the rats was recorded every three days; and their body weights were recorded every week. After the experiment, a concentration of 20% uratan solution (0.7 mL/100 g) was used, and the liver and bladder were removed after anesthesia.

### 2.2. Reagents and Antibodies

Sodium arsenite (Sigma, St. Louis, MO, USA; purity > 99.0%) and the NaAsO_2_ solution were prepared in sterile PBS. To ensure the toxicity of NaAsO_2_, it was prepared immediately before every experiment. MiRNA-125a-5p mimic and riboFectTM CP transfection reagent were purchased from Ribo (Guangzhou, China). Antibodies for TNF-α, IL-10, and IL-1β were purchased from Abclonal (Wuhan, China). Antibodies for VEGF, iNOS , Arg1, CD206 and IRF4 were purchased from Proteintech (Wuhan, China). The secondary antibodies were purchased from LICOR Biosciences (Lincoln, NE, USA).

### 2.3. Paraffin Embedding and Sectioning

The liver tissue was fixed with 4% paraformaldehyde for 72 h and dehydrated with 70% alcohol for 24 h. Gradient alcohol dehydration occurred for 1 h each time: we dehydrated with 100% alcohol for 30 min and repeated this three times. We soaked xylene three times, 15 min/time. For embedding, soft wax was used for 90 min and hard wax for 60 min, then it was stored at −20 °C. Paraffin sections 5 μm in size were prepared and roasted at 60 °C for 3–4 h. The bladder tissue was fixed with 4% paraformaldehyde for 48 h and dehydrated with 70% and 80% alcohol for 12 h; 90% alcohol for 1 h; and 95% alcohol for 30 min, then dehydrated with 100% alcohol for 20 min, and this was repeated twice. We soaked it in xylene for 10 min. Soft wax and hard wax were implanted for 60 min, respectively, and then treated with liver tissue.

### 2.4. Cell Culture

Human mononuclear THP-1 cells were purchased from the National Collection of Authenticated Cell Cultures (Shanghai, China) and cultured in RPMI 1640 medium (Bioind, Kibbutz Beit Haemek, Israel) containing 10% FBS (Bioind, Israel) and 1% penicillin (Bioind, Israel). The cells were cultured in a cell incubator containing 5% CO2 at 37 °C. Sodium arsenite was added to macrophages derived from THP-1 for 48 h (0, 2, 4, and 8 μM), and IL-4 (20 ng/mL) was used as the positive control. More than 3 parallel samples were set for each group of experiments.

### 2.5. The Establishment and Identification of the Macrophage Model

PMA (Sigma, St. Louis, MO, USA) was added to the cell suspension for 48 h and then removed. Accutase (Shanghai, China) was added and the cells were placed in an incubator for digestion for 5–10 min. After digestion, the cells were washed with precooled PBS and centrifuged to remove the supernatant. The cells were precipitated by PBS and incubated at 4 °C in the dark with antibody PE-CD11b (BioLegend, San Diego, CA, USA). For the precooled PBS resuspension cells, flow cytometry was used to detect the fluorescence value of the macrophage differentiation antigen and the percentage of CD11b. More than 3 parallel samples were set for each group of experiments.

### 2.6. Macrophage Viability

THP-1 cells in logarithmic growth phase were seeded in 96-well plates at a cell density of 1 × 10^4^/well with 100 μL of cell suspension per well. PMA was added and the cells were cultured for 48 h to induce macrophage differentiation. The medium was replaced with different arsenic concentrations (0, 2, 4, 8, 10, 12, and 14 μM) with 6 replicate wells per set. After 48 h of incubation, CCK8 reagent was added, 10 μL per well, and after 2 h of incubation, the absorbance value at 450 nm was determined by the microplate reader.

### 2.7. Flow Cytometry

CD86 (FITC) was prepared according to the flow cytometry antibody instructions, added to the digested M0 macrophages, and incubated at 4 °C in the dark for 30 min. After centrifugation, the cells were washed once with PBS. Subsequently, Triton was added and the samples were placed at room temperature for 10–15 min, centrifuged again, and CD206 antibody (APC) was added. After centrifugation and resuspension, the suspension was inhaled into the flow cytometry tube for analysis using a BD FACS Canto II instrument.

### 2.8. Cell Transfection

The has-miR-125a-5p overexpression model was constructed, and the has-miR-125a-5p mimic or miR-NC was transfected into cells. After induction of THP-1 into macrophages by PMA, the macrophages were washed twice with PBS and cultured in non-antibiotic complete RPMI 1640 medium. According to the instructions of the mimic RiboFectTM CP transfection reagent (Guangzhou, China), the corresponding transfection reagent was added into the six-well plate according to the system of 1.5 mL per well for 2 h. Then the corresponding concentration of NaAsO_2_ or IL-4 was added and the cells were incubated for 48 h.

### 2.9. Immunofluorescence Staining

The paraffin sections were placed on a heating platform and heated for 1–2 h. After xylene dewaxing, gradient alcohol dehydration, PBS cleaning, and 0.01 M sodium citrate solution antigen repair, the sections were washed 3 times in PBS and dried with filter paper. We dropped the sealing solution and placed it in a wet box at 37 °C for 30 min. Dilute primary antibody (Wuhan, China) was added and incubated overnight at 4 °C. The unbound primary antibody was removed by rewarming at 37 °C for 1 h and washing with PBS. The fluorescent secondary antibody (Beyotime, Shanghai, China) corresponding to the primary antibody was added and incubated in a wet box at 37 °C away from light. DAPI stain was added, incubated at room temperature and kept away from light, and PBS was washed to remove excess stain. The tablets were observed by fluorescence microscope and photographed. For cell immunofluorescence staining, we discarded the culture medium. We then cleaned with PBS, fixed with 4% paraformaldehyde, and incubated with sealing solution for 1 h, and staining was consistent with tissue sections.

### 2.10. RNA Extraction and Real-Time Quantitative PCR

Total RNA from cells was extracted using TRIzol reagent (TaKaRa, Dalian, China) and was reverse transcribed to cDNA using RT Reagent Kit (R223 or MR101) (Vazyme, Nanjing, China). Real-time quantitative PCR (RT-qPCR) of cDNA was performed on the Quant Studio 6 Flex QRT-PCR system (Life Technologies, Carlsbad, CA, USA) using SYBR qPCR Master Mix (Vazyme, Nanjing, China) or miRNA Universal qPCR Master Mix (Vazyme). The relative amount of mRNA normalized to GAPDH or U6 gene expression was calculated using the ΔΔCT method.

Specific forward and reverse primer sequences are shown in Table 1.

### 2.11. Western Blot

Tissue and cells were extracted by RIPA buffer. Concentrations of total protein were determined by the BCA Protein Assay Kit (Beyotime, Shanghai, China), according to the manufacturer’s instructions. Protein was separated by SDS-PAGE and transferred onto the PVDF membrane. Membranes were blocked and incubated with primary antibodies overnight at 4 °C. Membranes were then washed in PBST three times and further incubated with appropriate secondary antibodies for 1 h at room temperature. Signals were detected by the LI-COR Odyssey Infrared Imaging system.

### 2.12. Statistical Analysis

We used SPSS 26.0 statistical software for data analysis of the quantitative data, represented as mean ± standard deviation (SD). One-way analysis of variance (ANOVA) was used to compare the statistical difference between the experimental group and the control group, and the LSD test was used for pair-to-group comparison. A two-sided *p* value of <0.05 was considered to be statistically significant.

## 3. Results

### 3.1. Macrophages Polarize to M2 Type in Liver and Bladder Tissues of Arsenic-Exposed Rats

**Figure 1:**
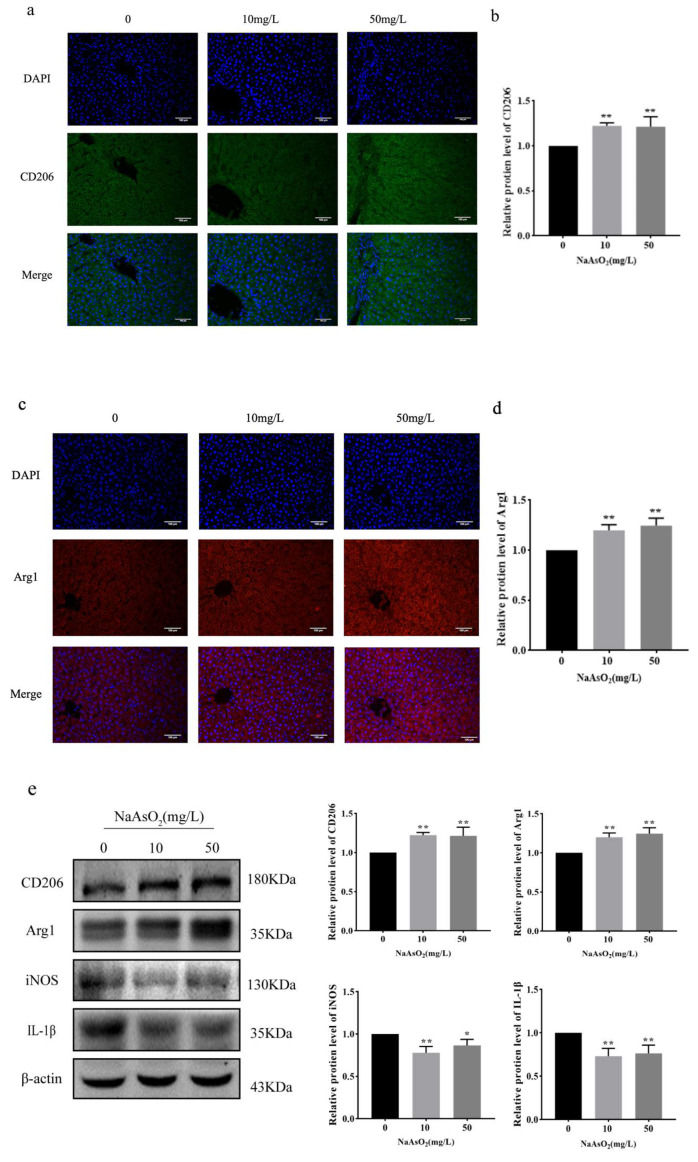

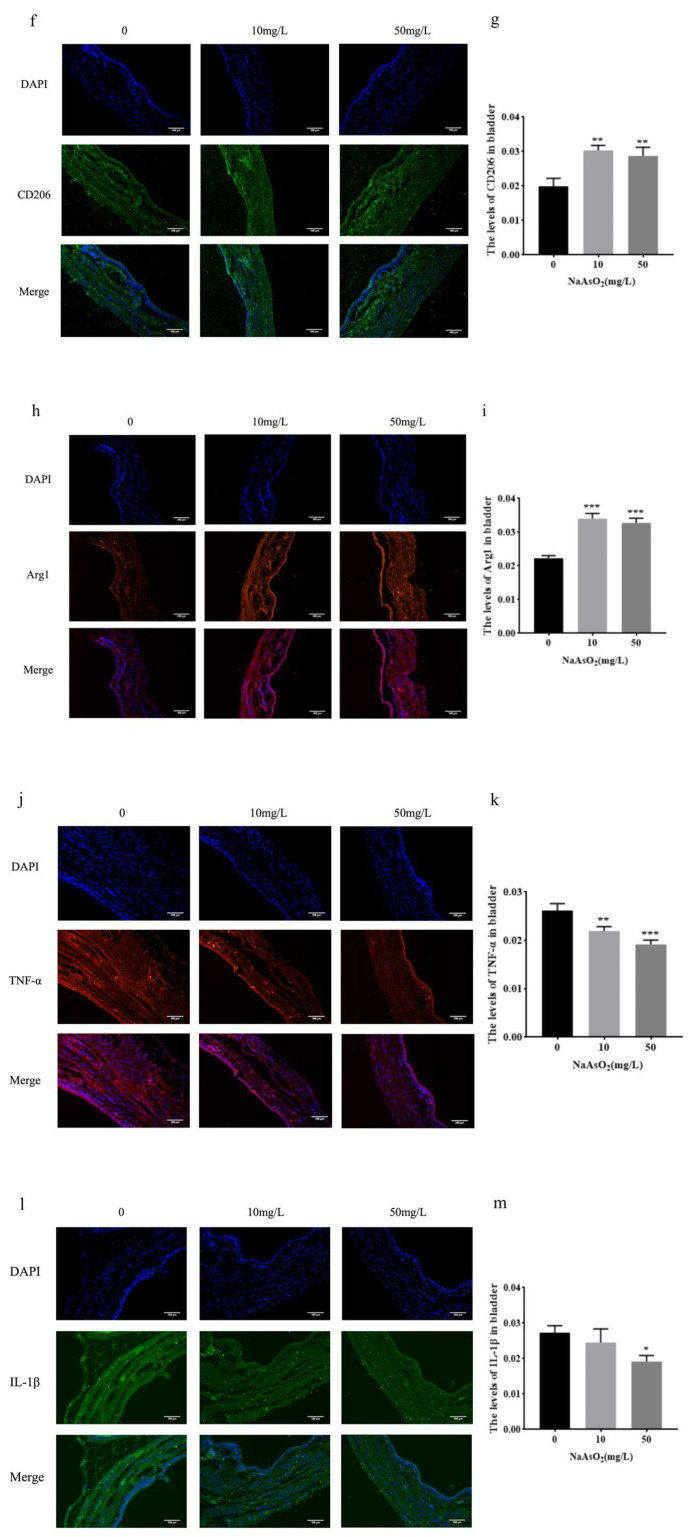
Polarization of macrophages in arsenic-exposed rat liver and bladder tissue. (**a**–**d**) Immunofluorescence of CD206 and Arg1 in liver tissues of rats (10×), bar = 100 μm. (**e**) Western Blot was used to detect the expression of CD206, Arg1, iNOS, and IL-1β. The original images can be found in Supplementary Materials. (**f**–**i**) Immunofluorescence of CD206 and Arg1 in bladder tissues of rats in each group (10×). (**j**–**m**) Immunofluorescence of inflammatory factors TNF-α and IL-1β in bladder tissues of rats (10×). * *p* < 0.05, ** *p* < 0.01, *** *p* < 0.001 vs. control group. Data are presented as mean ± SD.

A model of subchronic arsenic exposure in drinking water was established in rats, and the expression of marker proteins of M2 macrophages and inflammatory factors in the liver and bladder tissues of rats was observed to detect the effect of arsenic on the polarization of macrophages (Figure 1). The immunofluorescence staining showed that the expression of CD206 and Arg1 (hallmark M2 markers) in liver tissues was significantly higher than that in the control group after arsenic exposure (*p* < 0.01) (Figure 1a–d). The results of Western Blot showed that the expression of M2 macrophages was consistent with the results of immunofluorescence staining, and the inflammatory factors iNOS and IL-1β were significantly decreased compared to those in the control group (*p* < 0.05) (Figure 1e). Similar results were found in bladder tissue, with significantly higher expression levels of CD206 and Arg1 in the arsenic-exposed group than in the control group (*p* < 0.01) (Figure 1f–i). Instead, the expression levels of inflammatory factors TNF-α and IL-1β were significantly reduced in the arsenic-exposed group compared to those of the control group (*p* < 0.05) (Figure 1j–m).

### 3.2. NaAsO_2_ Induced the Expression of THP-1-Derived Macrophage-Related Genes In Vitro

**Figure 2:**
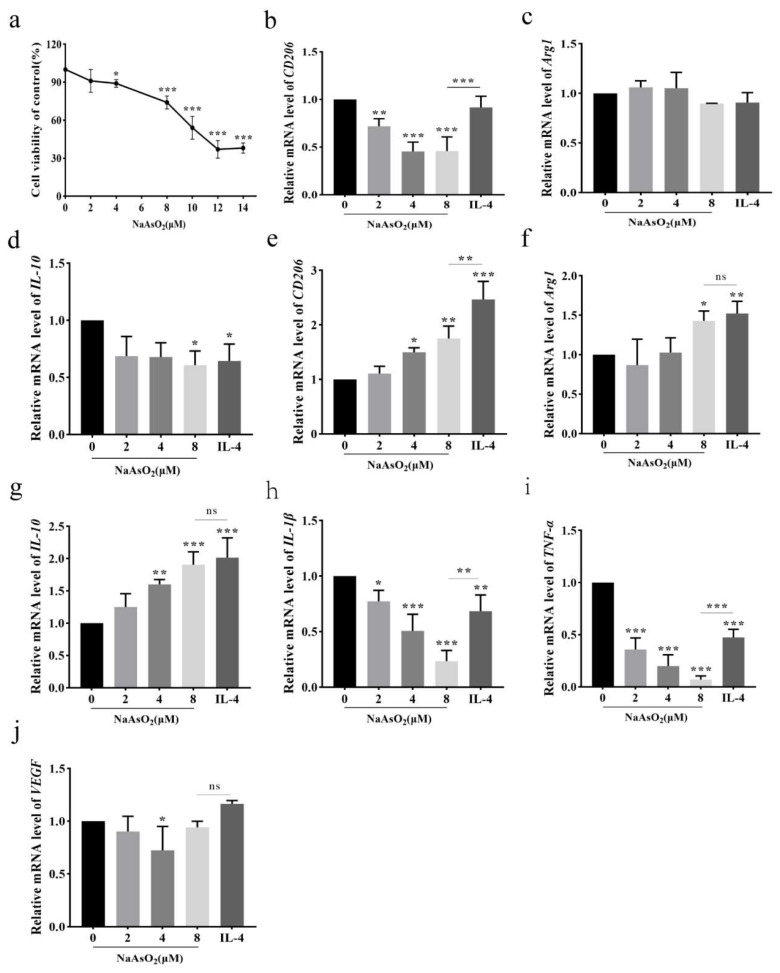
The viability and macrophage-related gene expression of THP-1-derived macrophages treated with different concentrations of NaAsO_2_. (**a**) Effect of different concentrations of NaAsO_2_ on the cell viability of THP-1-derived macrophages after 48 h treatment. (**b**–**d**) The mRNA expression of *CD206, Arg1*, and *IL-10* in THP-1-derived macrophages treated with different concentrations of NaAsO_2_ for 24 h. (**e**–**g**) The mRNA expressions of *CD206*, *Arg1*, and *IL-10* in THP-1-derived macrophages treated with different concentrations of NaAsO_2_ for 48 h. (**h**–**j**) The mRNA expression of *IL-1β*, *TNF-α*, and *VEGF* in THP-1-derived macrophages treated with different concentrations of NaAsO_2_ for 48 h compared with the control group. Data are presented as mean ± SD. * *p* < 0.05; ** *p* < 0.01; *** *p* < 0.001 vs. control group; ns: 8 μM vs. IL-4.

The effects of different concentrations of NaAsO_2_ on the activity of THP-1-derived macrophages were detected. Cell viability assays revealed dose-dependent cytotoxicity, with significant reductions observed at 4 μM NaAsO_2_ or higher (*p* < 0.05); the cell viability plummeted to lower than 50% at 10 μM NaAsO_2_ (Figure 2a). THP-1-derived macrophages were induced by different doses of NaAsO_2_ and treated for 24 h and 48 h. After 24 h treatment, *CD206* and *IL-10* mRNA levels were significantly decreased compared with the control group (*p* < 0.05; Figure 2b,d), while *Arg1* mRNA level did not change significantly (Figure 2c). Strikingly, prolonged 48 h exposure reversed this trend, inducing robust upregulation of *CD206*, *Arg1*, and *IL-10* (*p* < 0.05; Figure 2e–g). Concurrently, pro-inflammatory factors *IL-1β* and *TNF-α* were suppressed compared to controls (*p* < 0.05; Figure 2h,i), exhibiting significantly lower expression than IL-4-treated groups (*p* < 0.01). NaAsO_2_ failed to induce M2d-specific *VEGF* expression (*p* > 0.05; Fig 2j), suggesting selective regulation of M2a/M2c rather than angiogenesis-associated M2d polarization.

### 3.3. NaAsO_2_ Induced the Expression of THP-1-Derived Macrophage-Related Proteins

**Figure 3:**
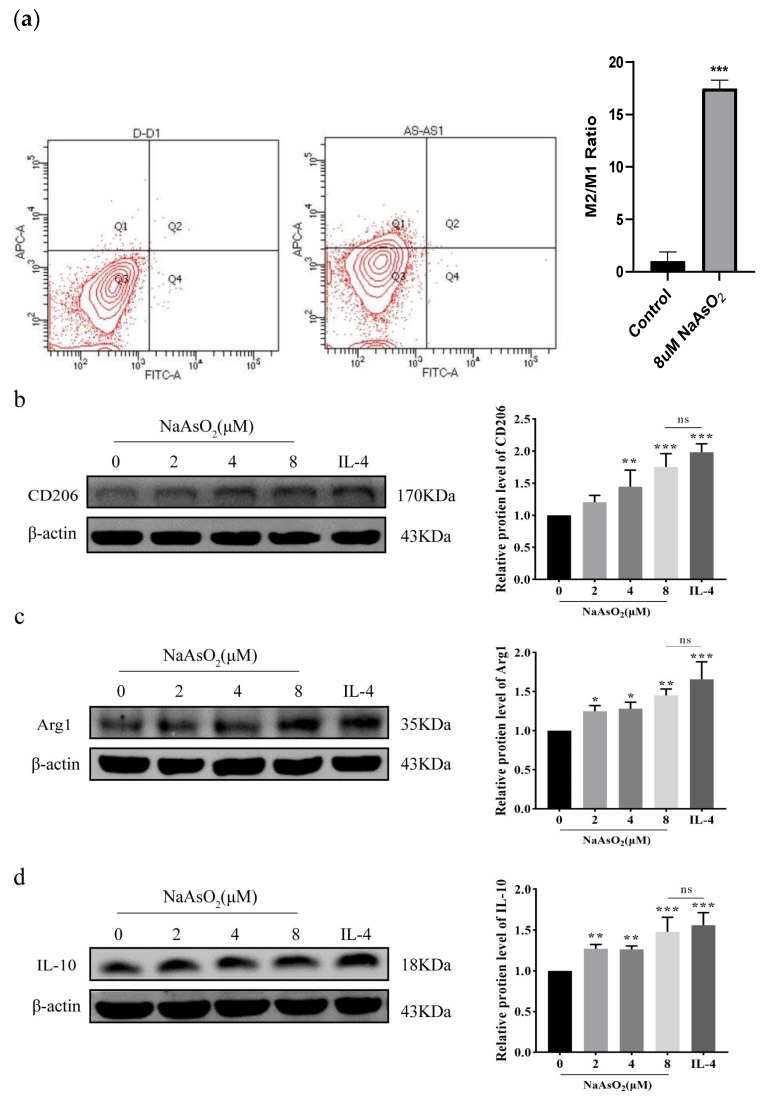

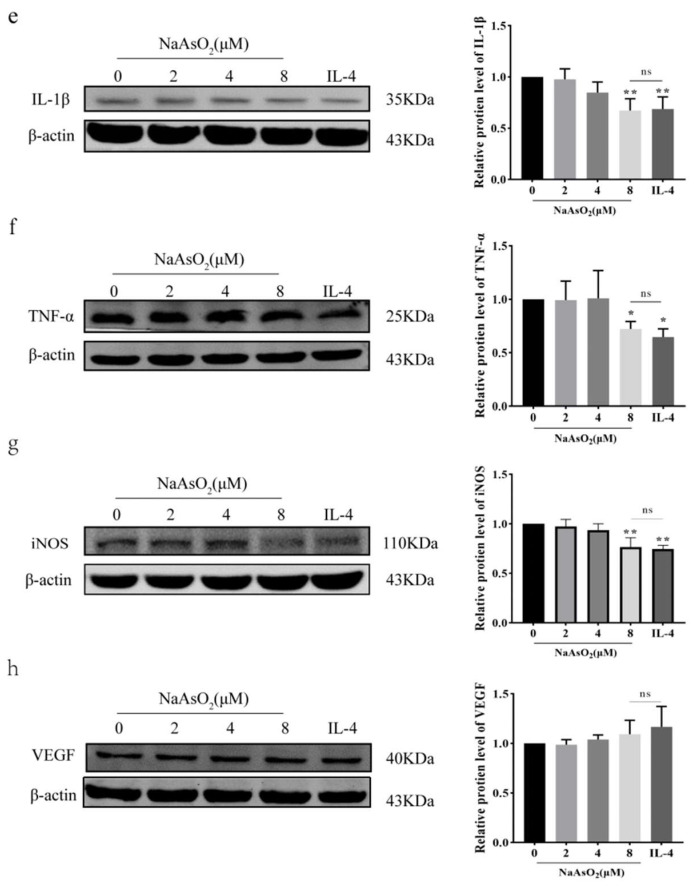
Expression results of related proteins in THP-1-derived macrophages treated with different concentrations of NaAsO_2_. (**a**) Flow cytometry showed that the M2/M1 ratio was significantly increased in THP-1-derived macrophages treated with NaAsO_2_. (**b**–**d**) The expression of macrophage markers CD206, Arg1, and IL-10 in M2 macrophages was detected by Western Blot. (**e**–**g**) The protein expression of inflammatory factors (IL-1β, TNF-α, and iNOS). (**h**) The protein expression of VEGF compared with the control group. The original images can be found in Supplementary Materials. Data are presented as mean ± SD. * *p* < 0.05; ** *p* < 0.01; *** *p* < 0.001 vs. control group; ns: 8 μM vs. IL-4.

Macrophages were treated with NaAsO_2_ for 48 h. Flow cytometric quantification of surface marker (CD206/CD86) ratios revealed that 48 h of NaAsO_2_ (8 μM) exposure increased the M2/M1 polarization index compared to untreated controls (*p* < 0.01), and macrophages were polarized toward M2 (Figure 3a). Western Blot analysis corroborated these changes in polarization, demonstrating significant upregulation of M2 markers in NaAsO_2_-treated macrophages: CD206, Arg1, and IL-10 (*p* < 0.05 vs. control; Figure 3b–d). And the effects paralleled IL-4-induced M2 polarization (*p* > 0.05), indicating comparable efficacy between NaAsO_2_ and cytokine-driven polarization. Along with M2 activation, NaAsO_2_ treatment suppressed pro-inflammatory factors, reducing IL-1β, TNF-α, and iNOS protein levels compared with controls (*p* < 0.05; Figure 3e–g), and there was no significant difference between IL-4 treatment groups. VEGF protein expression was subsequently detected, and there was no significant difference between the treatment groups and the control group (Figure 3h).

### 3.4. NaAsO_2_ Suppresses miR-125a-5p Expression in Polarized Macrophages

**Figure 4:**
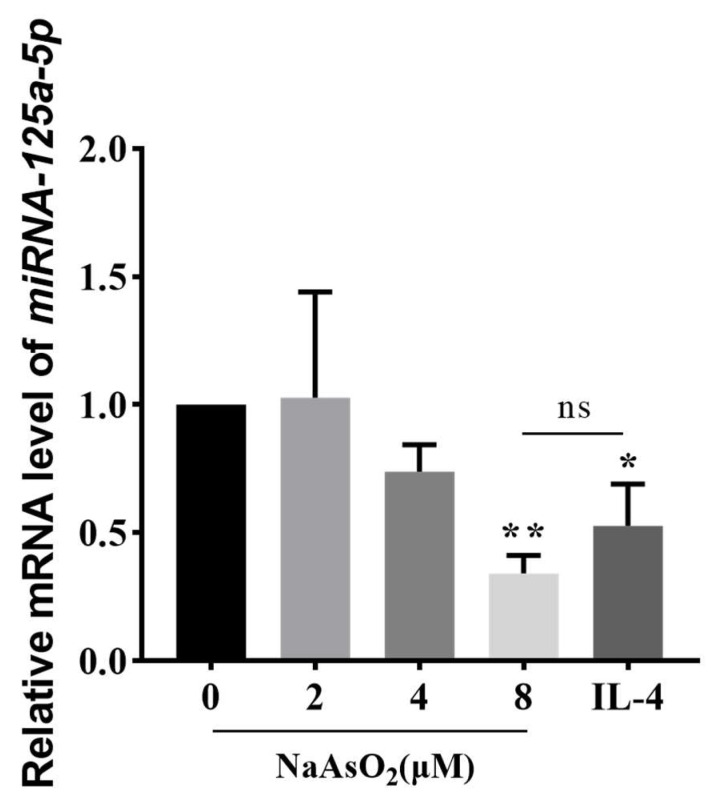
The mRNA expression of miR-125a-5p in THP-1-derived macrophages induced by different concentrations of NaAsO_2_ compared with the control group. Data are presented as mean ± SD. * *p* < 0.05; ** *p* < 0.01 vs. control group; ns: 8 μM vs IL-4.

The expression of miR-125a-5p in cells treated with different concentrations of NaAsO_2_ or IL-4 for 48 h was detected by stem-loop RT-qPCR (Figure 4). Compared with the control group, mRNA levels of miR-125a-5p in the 8 μM NaAsO_2_ and IL-4 treatment groups were significantly decreased (*p* < 0.05), and there was no statistical difference between the two treatment groups. This coordinated miR-125a-5p attenuation aligns with the observed M2 polarization phenotype, implicating epigenetic regulation in arsenic-driven immune reprogramming.

### 3.5. Bioinformatics Prediction of MiR-125a-5p Target Genes

**Figure 5:**
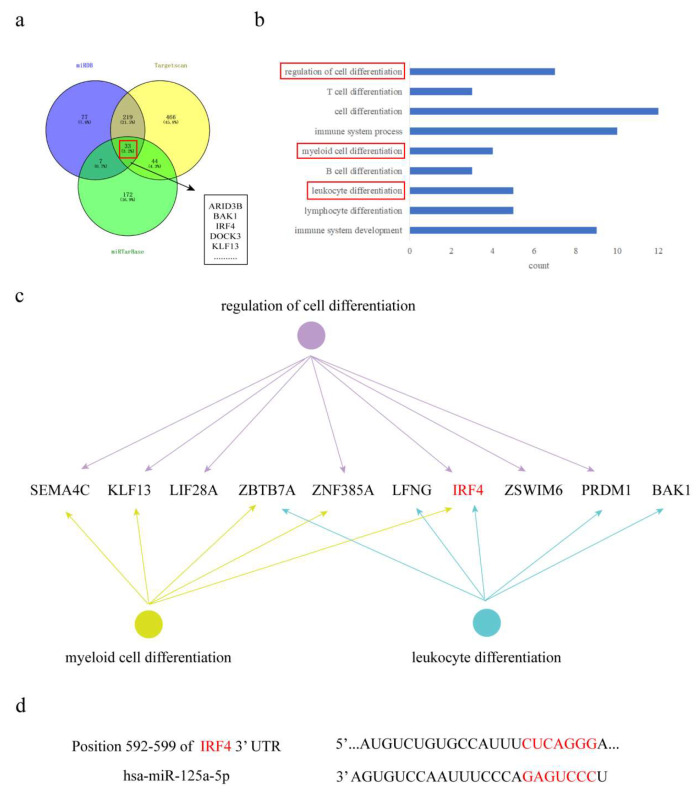
Prediction results of miR-125a-5p target genes. (**a**) Venn diagram shows that three miRNA binding sites were used to predict miRDB, Targetscan, and miRTarBase finding miR-125a-5p target proteins. (**b**,**c**) DAVID database GO enrichment analysis and visual display. (**d**) Targetscan database predicting binding sites of miR-125a-5p and IRF4.

The target sites of miR-125a-5p were screened by miRDB (https://www.mirdb.org (accessed on 1 June 2022)), Targetscan (https://www.targetscan.org (accessed on 1 June 2022)), and miRTarBase (https://mirtarbase.cuhk.edu.cn (accessed on 1 June 2022)). Genes with higher binding site scores were selected, and 33 target genes were screened by intersection (Figure 5a). Gene Ontology (GO) enrichment via the DAVID database revealed IRF4 as a critical regulator of immune cell differentiation (Figure 5b,c). TargetScan predicted a conserved binding site between miR-125a-5p and IRF4 3′-UTR (position 592–599; Figure 5d), establishing their direct regulatory relationship.

### 3.6. MiR-125a-5p/IRF4 Axis Mediates Arsenic-Driven Macrophage Polarization via Epigenetic Regulation

**Figure 6:**
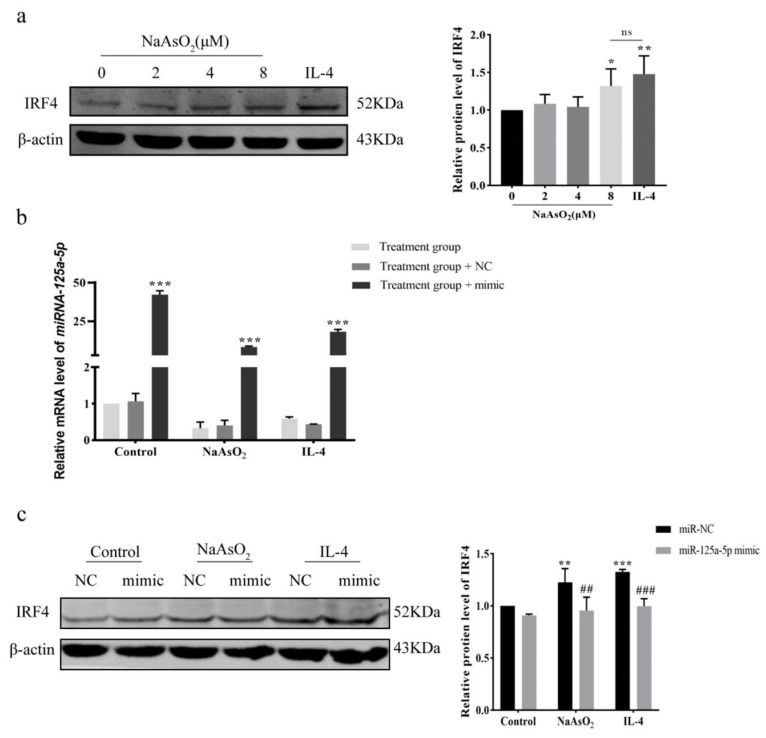
MiR-125a-5p overexpression reverses NaAsO_2_-induced IRF4 upregulation in macrophages. (**a**) Western Blot analysis of IRF4 protein expression in THP-1-derived macrophages induced by different concentrations of NaAsO_2_. (**b**) RT-qPCR confirmed successful miR-125a-5p overexpression with mimic transfection. (**c**) Western Blot was used to detect the expression of IRF4 protein in each treatment group after transfection with miR-125a-5p NC or mimic. The original images can be found in Supplementary Materials. * *p* < 0.05, ** *p* < 0.01, *** *p* < 0.001 vs. control/control -miR-NC group; ## *p* < 0.01, ### *p* < 0.001 vs. NaAsO_2_/IL-4-miR-NC group. Data = mean ± SD (n = 3); ns: not significant.

The levels of the miR-125a-5p target protein IRF4 were detected by Western Blot, and the results showed that IRF4 was significantly increased in the 8 μM NaAsO_2_ and IL-4 treatment groups compared with the control group (*p* < 0.05) (Figure 6a). Transfection efficiency was verified after 48 h of transfection with miR-125a-5p NC or mimic. The mRNA levels of miR-125a-5p were significantly increased in control, NaAsO_2_-, or IL-4-treatment groups transfected with miR-125a-5p mimic compared with the corresponding groups without mimic transfection (*p* < 0.001), which confirmed that miR-125a-5p overexpression had been successfully constructed (Figure 6b). Western Blot was used to detect the expression level of IRF4 protein after transfection of miR-125a-5p. MiR-125a-5p mimic transfection abolished NaAsO_2_/IL-4-induced IRF4 upregulation (*p* < 0.01; Figure 6c), while control-miR-NC retained IRF4 elevation (*p* > 0.05). This suppression confirmed miR-125a-5p as a master regulator of IRF4 expression.

### 3.7. MiR-125a-5p Overexpression Inhibited NaAsO_2_-Induced M2 Polarization via IRF4 Suppression

**Figure 7:**
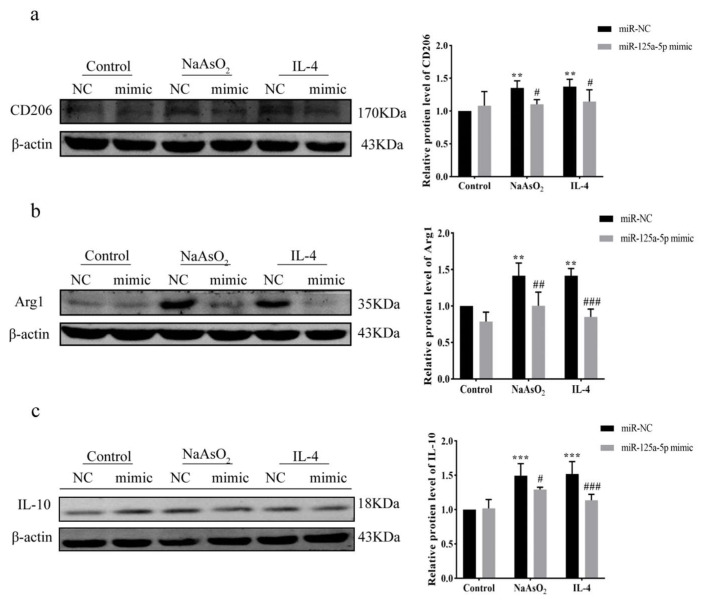
Expression results and quantitative statistics of M2 macrophage-related proteins in each treatment group after miR-125a-5p overexpression. (**a**–**c**) Western Blot was used to detect the expression of CD206, Arg1, and IL-10 in each treatment group after transfection with miR-125a-5p NC or mimic. The original images can be found in Supplementary Materials. ** *p* < 0.01, *** *p* < 0.001, vs. the control-miR-NC group; # *p* < 0.05; ## *p* < 0.01; ### *p* < 0.001, vs. NaAsO_2_ or IL-4-miR-NC group. Data are presented as mean ± SD.

Western Blot was used to detect the expression levels of related proteins in M2 macrophages transfected with miR-125a-5p NC or mimic. As shown in the result, CD206, Arg1, and IL-10 protein levels in NaAsO_2_-miR-NC and IL-4-miR-NC groups were significantly increased compared with the control-miR-NC group (*p* < 0.01); there was no statistical difference between the control-miR-NC and control-miR-mimic groups. However, CD206, Arg1, and IL-10 protein levels in the NaAsO_2_-miR-mimic and IL-4-miR-mimic groups were significantly decreased after transfection compared with those of the NaAsO_2_-miR-NC and IL-4-miR-NC groups (*p* < 0.05) (Figure 7a–c).

### 3.8. MiR-125a-5p Overexpression Restores Inflammation via Attenuating NaAsO_2_-Mediated Suppression

**Figure 8:**
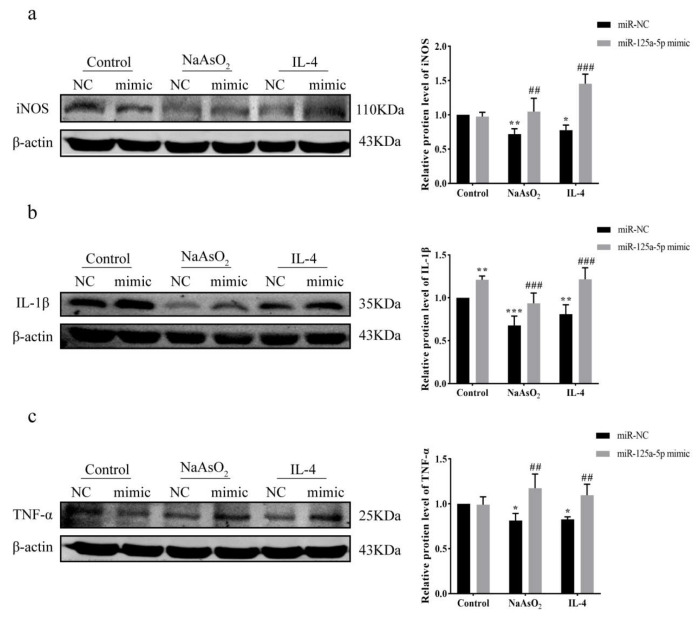
Expression of inflammatory-factor-related proteins after MiR-125a-5p overexpression. (**a**) Western Blot was used to detect the expression of iNOS after transfection with miR-125a-5p NC or mimic. (**b**) Western Blot was used to detect the expression of IL-1β after transfection with miR-125a-5p NC or mimic. (**c**) Western Blot was used to detect the expression of TNF-α in each treatment group after transfection with miR-125a-5p NC or mimic. The original images can be found in Supplementary Materials. Data are presented as mean ± SD (n = 3). * *p* < 0.05, ** *p* < 0.01, *** *p* < 0.001, vs. control-miR-NC; ## *p* < 0.01, ### *p* < 0.001, vs. NaAsO_2_/IL-4-miR-NC.

Western Blot was used to detect the expression levels of inflammatory-factor-related proteins after transfection with miR-125a-5p NC or mimic. As shown in Figure 8a–c, the iNOS, IL-1β, and TNF-α protein levels in the NaAsO_2_-miR-NC and IL-4-miR-NC groups were significantly decreased compared with the control-miR-NC group (*p* < 0.05). The levels of IL-1β in the control-miR-mimic group were significantly higher than those in the control-miR-NC group (*p* < 0.01); there was no significant difference in iNOS and TNF-α protein levels between the control-miR-NC and control-miR-mimic groups. Crucially, mimic transfection reversed NaAsO_2_/IL-4-induced suppression of inflammatory factors.

## 4. Discussion

Arsenic is a class I carcinogen confirmed by the International Center for Research on Cancer (IARC). It exerts systemic toxicity through chronic exposure, driving immunotoxicity and carcinogenesis via mechanisms that remain incompletely defined. Our study reveals an epigenetic pathway by which arsenic disrupts macrophage polarization—a critical determinant of immune homeostasis and tumor microenvironment formation.

The imbalance between anti-inflammatory and pro-inflammatory effects disrupts immune function in the body, and macrophage polarization balance (M1/M2) controls inflammatory responses in different pathophysiological contexts. In patients with rheumatoid arthritis (RA), M1/Th1 becomes activated, leading to severe osteoclasts, erosion, and progressive joint destruction. The activation of M2/Th2 response enables the release of growth factors and cytokines in the anti-inflammatory process, thus relieving the symptoms of RA [14]. In models of atherosclerosis, promoting polarization toward the M1 pro-inflammatory phenotype can accelerate the progression of atherosclerosis, while reducing the polarization of macrophage inflammation can improve the progression of atherosclerosis [15,16,17]. In contrast, during HIV infection, macrophages are the most important reservoir of HIV and become resistant. Both M1 and M2 macrophage polarization can inhibit HIV infection, while the M2 antiviral program is relatively weak. However, during the transition stage of HIV infection, the M1 polarization switch occurs in circulating monocytes, which is speculated to be the establishment process of chronic latent infection [18,19]. In an aging population, the M2 macrophage polarization phenomenon is found, and the chemotaxis, antigen presenting ability, and phagocytosis ability are weakened. It is suggested that polarization towards M2 macrophages increases the susceptibility of the elderly to diseases. Our study found that sodium arsenite can break the balance of macrophages, resulting in a tendency toward M2 polarization, which can weaken the anti-infection ability of the body and cause immune dysfunction.

By establishing a rat model of subchronic arsenic exposure in drinking water, we found that the level of M2 macrophages increased and that of M1 macrophages decreased in liver and bladder tissues. We speculated that arsenic might induce M2 polarization of macrophages, which may be partly due to the change in tissue-resident macrophages. Macrophages can reside in most tissues and maintain their own state for a long time. Tissue-resident macrophages respond to the outside world to regulate the dynamic balance of tissues. By in vitro experiments, we compared the polarization types of THP-1-derived macrophages treated with different concentrations of sodium arsenite at 24 h and 48 h. IL-4 treatment was used as a positive control for M2 polarization. It was found that macrophages were mainly polarized into M2 macrophages after 48 h treatment. After induction of arsenic, macrophages may reduce their ability to fight against bacteria and viruses, resulting in immune disorders, and M2 macrophages also play a promotional role in inducing tumors.

According to different activation stimuli, M2 macrophages can be further divided into M2a, M2b, M2c, and M2d subtypes, which perform their respective functions [20,21,22]. Among them, biological functional factors produced by M2a are beneficial to wound healing, debris removal, and the end of the inflammatory response [23]. Induced by immune complex engagement and Toll-like receptor (TLR) activation, M2b macrophages are identified by increased production of the anti-inflammatory cytokines IL-10 and C-C motif ligand (CCL)-1, as well as a notable ability to present antigens, as demonstrated by increased expression of CD86 [24]. M2c macrophages exhibit strong anti-inflammatory activity to inhibit apoptotic cells. M2d macrophages induce the secretion of IL-10 and vascular endothelial growth factor (VEGF), a phenotype that contributes to tumor mass survival and is thought to be caused by tumor secretion factors [25]. Huang et al. [26] examined the THP-1 cell supernatant under low-dose arsenic treatment for 24 h and found that arsenic increased M2c-associated cytokine IL-10 and chemokine CCL-16, and found arsenic-treated macrophages promoted A549 lung epithelial cell invasion and migration. In this study, we examined the protein in cells treated with arsenic for 48 h. Comparing the protein expression of VEGF in the arsenic-exposed group and the control group, the results indicated no statistical difference. Combined with the results, we guessed that the polarization type of macrophages induced by sodium arsenite might be M2a type. However, during chronic arsenic exposure, this typically reparative M2a polarization may exert detrimental effects. Through the suppression of immune surveillance and induction of immunosuppression, M2a polarization could inadvertently promote tumor initiation and progression in tissues targeted by arsenic, notably the liver and bladder.

Mechanistically, we identified miR-125a-5p suppression as a pivotal epigenetic trigger. MicroRNA plays an epigenetic regulatory role in organisms. The miR-125 family targets many genes, such as transcription factors and members of the Bcl-2 family, whose abnormality may lead to abnormal cell proliferation, metastasis, and even cancer formation [27]. In addition, the miR-125 family is also involved in immune host defense processes. IRF-4 is a member of the interferon regulatory factors (IRFs) family. IRFs play a key role in many aspects, such as monocyte hematopoietic development, macrophage polarization, phenotypic transformation, and functional regulation [28]. While IRF4 is recognized as a master regulator of M2 polarization [29], its upstream control by miRNAs in arsenic toxicity is unknown. In our study, it was found that the expression of miR-125a-5p in M2-type macrophages induced by sodium arsenite was significantly reduced. Bioinformatic prediction and experimental validation confirmed IRF4, a master transcriptional regulator of M2 polarization, as a direct target of miR-125a-5p. NaAsO_2_ significantly downregulated miR-125a-5p expression, with IRF4 upregulation occurring. MiR-125a-5p overexpression reversed NaAsO_2_-induced M2 polarization by suppressing IRF4, restoring M1-associated proteins.

M1 and M2 macrophage polarization is a complex process of the interaction of several factors. The balance between IRF4 and IRF5 may be the main determinant of the polarization of M1 and M2 macrophages. IRF4 and IRF5 combine competitively with MyD88 [30]. Studies have shown that IRF5 and IRF4 correspond to pro-inflammatory and anti-inflammatory features in microglia after stroke, and IRF5-IRF4 signals form a regulatory axis to balance pro-inflammatory and anti-inflammatory activation in microglia. When the expression of IRF5 is suppressed, the expression level of IRF4 is increased, and when the expression of IRF4 is downregulated, the anti-inflammatory cytokines produced by microglia also have corresponding changes, which also affect the infiltration of peripheral immune cells and the circulation level of cytokines [31]. Our findings suggest arsenic disrupts this balance by specifically elevating IRF4 via miR-125a-5p suppression. The overexpression of miR-125a-5p may enable the transformation of sodium arsenite-induced M2-type macrophages into M1-type macrophages, but whether it is through the regulation of the IRF5-IRF4 axis or the participation of other polarization pathways (potentially involving pathways like PI3K/AKT, Notch signal, and epigenetics) [10,32,33,34] remains to be explored. We noted that the classical effects of arsenic, such as oxidative stress and DNA damage, may also be involved in immunomodulation. The epigenetic mechanisms revealed in this study provide a molecular explanation for arsenic-induced M2-type polarization in macrophages, but the potential cross-talk between it and oxidative stress and DNA damage remains to be further explored.

In conclusion, we reveal a novel epigenetic pathway by which arsenic disrupts immune homeostasis: suppression of miR-125a-5p derepresses IRF4, driving aberrant M2 macrophage polarization. This shift fosters an immunosuppressive TME conducive to arsenic-associated carcinogenesis. Whereas TAMs, specifically the M2 phenotype, have an established role in promoting tumor progression, angiogenesis, and metastasis. In recent years, macrophages have gradually become the research target of anti-tumor therapy, which has strong practical value and significance [35]. Our findings highlight the miR-125a-5p/IRF4 axis as a promising therapeutic target. Strategies to restore miR-125a-5p function or inhibit IRF4 could potentially counteract arsenic-induced immunosuppression and hinder tumor development. While TAM-targeting therapies are actively explored [36,37], our work provides a specific mechanistic foundation for developing interventions tailored to arsenic-driven malignancies.

## 5. Conclusions

Our study elucidates a novel epigenetic mechanism underlying arsenic-induced immune dysfunction and carcinogenesis. We demonstrate that chronic exposure to sodium arsenite disrupts macrophage polarization homeostasis by suppressing miR-125a-5p expression, leading to the derepression and upregulation of its target, IRF4. This miR-125a-5p/IRF4 axis drives a significant shift towards M2 macrophage polarization. This aberrant M2 polarization fosters an immunosuppressive microenvironment characterized by impaired anti-pathogen defenses and diminished immune surveillance.

## Figures and Tables

**Table 1:** PCR primer sequence.

Gene	Forward Primer (5′-3′)	Reverse Primer (5′-3′)
*CD206*	GCAAAGTGGATTACGTGTCTTG	CTGTTATGTCGCTGGCAAATG
*Arg1*	GTCTGTGGGAAAAGCAAGCG	CACCAGGCTGATTCTTCCGT
*IL-10*	AAGACCCAGACATCAAGGCG	AGGCATTCTTCACCTGCTCC
*IL-1β*	CTGCTCTGGGATTCTCTTCAG	ATCTGTTTAGGGCCATCAGC
*TNF-α*	ACTTTGGAGTGATCGGCC	GCTTGAGGGTTTGCTACAAC
*VEGF*	AGGGCAGAATCATCACGAAG	GGATGGCTTGAAGATGTACTCG
*GAPDH*	TGTTGCCATCAATGACCCCTT	CTCCACGACGTACTCAGCG
*miR-125a-5p*	GCGTCCCTGAGACCCTTTAAC	AGTGCAGGGTCCGAGGTATT
*U6*	AGAGAAGATTAGCATGGCCCCTG	AGTGCAGGGTCCGAGGTATT
*miR-125a-5p Stem-Loop primer*	GTCGTATCCAGTGCAGGGTCCGAGGTAT TCGCACTGGATACGACTCACAG	
*U6 Stem-Loop primer*	GTCGTATCCAGTGCAGGGTCCGAGGTATTCGCACTGGATACGACAAAATATG	

## Data Availability

The original contributions presented in this study are included in the article/Supplementary Material. Further inquiries can be directed to the corresponding authors.

## Supplementary Materials

The following supporting information can be downloaded at https://www.mdpi.com/article/10.3390/biom15111630/s1: Figure S1: Morphological changes of THP-1 cells treated with PMA and expression of macrophage marker CD11b. And Western Blot Original Images.

## References

[1] Hughes M.F. (2006). Biomarkers of exposure: A case study with inorganic arsenic. Environ. Health Perspect..

[2] Rahaman M.S., Yamasaki S., Hossain K.F.B., Hosokawa T., Saito T., Kurasaki M. (2020). Effects of curcumin, D-pinitol alone or in combination in cytotoxicity induced by arsenic in PC12 cells. Food Chem. Toxicol..

[3] Baris D.R., Waddell R., Freeman L.B., Schwenn M., Silverman D. (2016). Elevated Bladder Cancer in Northern New England: The Role of Drinking Water and Arsenic. J. Natl. Cancer Inst..

[4] Kim T.H., Seo J.W., Hong Y.S., Song K.H. (2017). Case-control study of chronic low-level exposure of inorganic arsenic species and non-melanoma skin cancer. J. Dermatol..

[5] Mantovani A., Sica A., Sozzani S., Allavena P., Vecchi A., Locati M. (2004). The chemokine system in diverse forms of macrophage activation and polarization. Trends Immunol..

[6] Yunna C., Mengru H., Lei W., Weidong C. (2020). Macrophage M1/M2 polarization. Eur. J. Pharmacol..

[7] Wang N., Liang H., Zen K. (2014). Molecular mechanisms that influence the macrophage m1-m2 polarization balance. Front. Immunol..

[8] Essandoh K., Li Y., Huo J., Fan G.C. (2016). MiRNA-Mediated Macrophage Polarization and its Potential Role in the Regulation of Inflammatory Response. Shock.

[9] Graff J.W., Dickson A.M., Clay G., McCaffrey A.P., Wilson M.E. (2012). Identifying functional microRNAs in macrophages with polarized phenotypes. J. Biol. Chem..

[10] Zhao J., Huang F., He F., Gao C., Liang S., Ma P., Dong G., Han H., Qin H. (2016). Forced Activation of Notch in Macrophages Represses Tumor Growth by Upregulating miR-125a and Disabling Tumor-Associated Macrophages. Cancer Res..

[11] Huang H.C., Yu H.R., Huang L.T., Huang H.C., Chen R.F., Lin I.C., Ou C.Y., Hsu T., Yang K. (2012). MiRNA-125b regulates TNF-α production in CD14+ neonatal monocytes via post-transcriptional regulation. J. Leukoc. Biol..

[12] Chaudhuri A.A., So Y.L., Sinha N., Gibson W., Baltimore D. (2011). MicroRNA-125b potentiates macrophage activation. J. Immunol..

[13] Wang J.K., Wang Z., Li G. (2019). MicroRNA-125 in immunity and cancer. Cancer Lett..

[14] Chang J., Tang C. (2024). The role of macrophage polarization in rheumatoid arthritis and osteoarthritis: Pathogenesis and therapeutic strategies. Int. Immunopharmacol..

[15] Babaev V.R., Hebron K.E., Wiese C.B., Toth C.L., Ding L., Zhang Y., May J.M., Fazio S., Vickers K.C., Linton M.F. (2014). Macrophage deficiency of Akt2 reduces atherosclerosis in Ldlr null mice. J. Lipid Res..

[16] Barrett T.J. (2020). Macrophages in Atherosclerosis Regression. Arter. Thromb. Vasc. Biol..

[17] Wang S., Yang S., Chen Y., Li R., Han S., Kamili A., Wu Y., Zhang W. (2022). Ginsenoside Rb2 Alleviated Atherosclerosis by Inhibiting M1 Macrophages Polarization Induced by MicroRNA-216a. Front. Pharmacol..

[18] Herbein G., Varin A. (2010). The macrophage in HIV-1 infection: From activation to deactivation?. Retrovirology.

[19] Sica A., Erreni M., Allavena P., Porta C. (2015). Macrophage polarization in pathology. Cell Mol. Life Sci..

[20] Rees P.A., Greaves N.S., Baguneid M., Bayat A. (2015). Chemokines in Wound Healing and as Potential Therapeutic Targets for Reducing Cutaneous Scarring. Adv. Wound Care.

[21] Abdelaziz M.H., Abdelwahab S.F., Wan J., Cai W., Huixuan W., Jianjun C., Kumar K.D., Vasudevan A., Sadek A., Su Z. (2020). Alternatively activated macrophages; a double-edged sword in allergic asthma. J. Transl. Med..

[22] Hesketh M., Sahin K.B., West Z.E., Murray R.Z. (2017). Macrophage Phenotypes Regulate Scar Formation and Chronic Wound Healing. Int. J. Mol. Sci..

[23] White M.J., Gomer R.H. (2015). Trypsin, Tryptase, and Thrombin Polarize Macrophages towards a Pro-Fibrotic M2a Phenotype. PLoS ONE.

[24] Xue J.D., Gao J., Tang A.F., Feng C. (2024). Shaping the immune landscape: Multidimensional environmental stimuli refine macrophage polarization and foster revolutionary approaches in tissue regeneration. Heliyon.

[25] Anders C.B., Lawton T.M.W., Ammons M.C. (2019). Metabolic immunomodulation of macrophage functional plasticity in nonhealing wounds. Curr. Opin. Infect. Dis..

[26] Hung C.H., Hsu H.Y., Chiou H.Y., Tsai M.Y., You H.Y., Lin Y.C., Liao W.T., Lin Y.C. (2022). Arsenic Induces M2 Macrophage Polarization and Shifts M1/M2 Cytokine Production via Mitophagy. Int. J. Mol. Sci..

[27] Sun Y.M., Lin K.Y., Chen Y.Q. (2013). Diverse functions of miR-125 family in different cell contexts. J. Hematol. Oncol..

[28] Chistiakov D.A., Myasoedova V.A., Revin V.V., Orekhov A.N., Bobryshev Y.V. (2018). The impact of interferon-regulatory factors to macrophage differentiation and polarization into M1 and M2. Immunobiology.

[29] Khayati S., Dehnavi S., Sadeghia M., Afshari J.T., Esmaeili S.A., Mohammadi M. (2023). The potential role of miRNA in regulating macrophage polarization. Heliyon.

[30] Günthner R., Anders H.J. (2013). Interferon-regulatory factors determine macrophage phenotype polarization. Mediat. Inflamm..

[31] Mamuna A.A., Chauhana A., Qia S., Ngwaa C., Xua Y., Sharmeena P., Hazenb A.L., Lia J., Aronowskia J.A., McCullougha L.D. (2020). Microglial IRF5-IRF4 regulatory axis regulates neuroinflammation after cerebral ischemia and impacts stroke outcomes. Proc. Natl. Acad. Sci. USA.

[32] Fang H., Yang M., Pan Q., Jin H., Li H., Wang R., Wang Q., Zhang J. (2021). MicroRNA-22-3p alleviates spinal cord ischemia/reperfusion injury by modulating M2 macrophage polarization via IRF5. J. Neurochem..

[33] Liang C., Tang Y., Gao X., Lei N., Luo Y., Chen P., Duan S., Cao Y., Yang Y., Zhang Y. (2023). Depression Exacerbates Dextran Sulfate Sodium-Induced Colitis via IRF5-Mediated Macrophage Polarization. Dig. Dis. Sci..

[34] Sasaki K., Terker A.S., Pan Y., Li Z., Harris R.C. (2021). Deletion of Myeloid Interferon Regulatory Factor 4 (Irf4) in Mouse Model Protects against Kidney Fibrosis after Ischemic Injury by Decreased Macrophage Recruitment and Activation. J. Am. Soc. Nephrol..

[35] Boutilier A.J., Elsawa S.F. (2021). Macrophage Polarization States in the Tumor Microenvironment. Int. J. Mol. Sci..

[36] Pan Y., Yu Y., Wang X., Zhang T. (2020). Tumor-Associated Macrophages in Tumor Immunity. Front. Immunol..

[37] Chen D., Zhang X., Li Z., Zhu B. (2021). Metabolic regulatory crosstalk between tumor microenvironment and tumor-associated macrophages. Theranostics.

