# Supplementary material for: The miR-125a-5p/IRF4 Axis Mediates Sodium Arsenite-Induced M2 Macrophage Polarization
Source: Biomolecules. 2025 Nov 20;15(11):1630. doi: 10.3390/biom15111630 (PMC12649919; doi:10.3390/biom15111630)
Supplement: Supplementary file 1 [file biomolecules-15-01630-s001.zip › biomolecules-3939521-original image.pdf]

Western Blot original data

The miR-125a-5p/IRF4 Axis Mediates Sodium Arsenite-Induced M2 Macrophage Polarization

Fig1 e

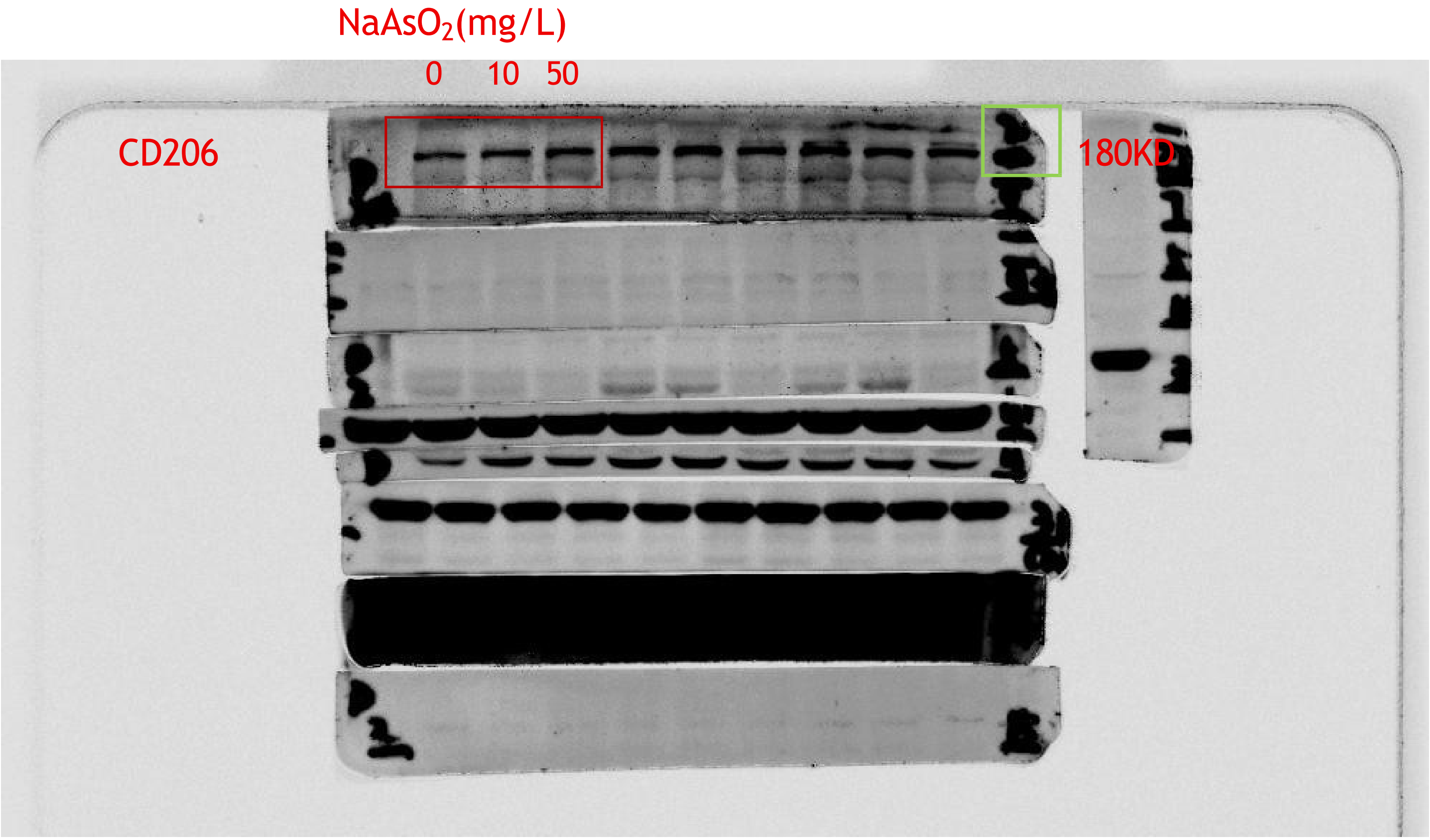

Fig1e Arg1

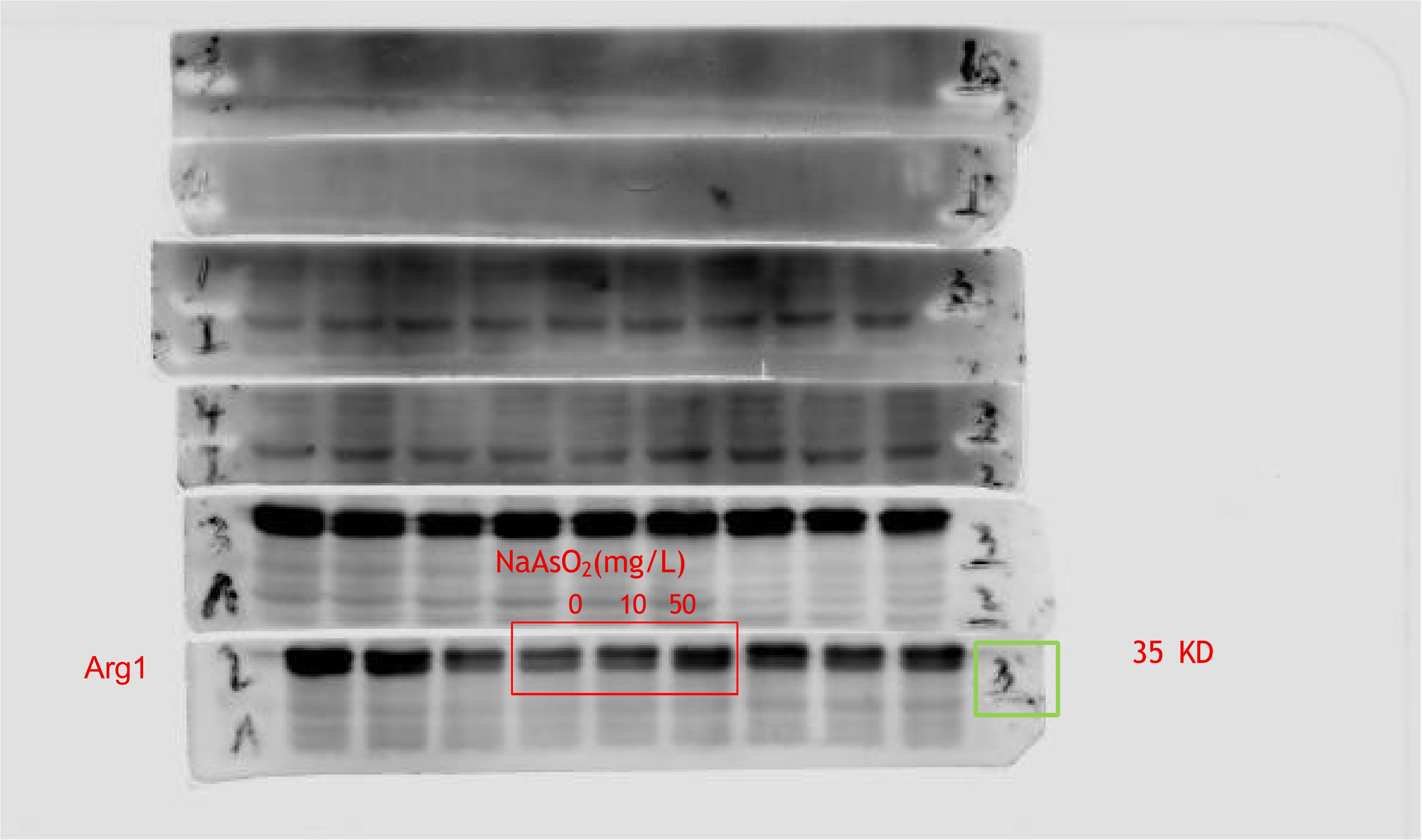

Fig1 e iNOS

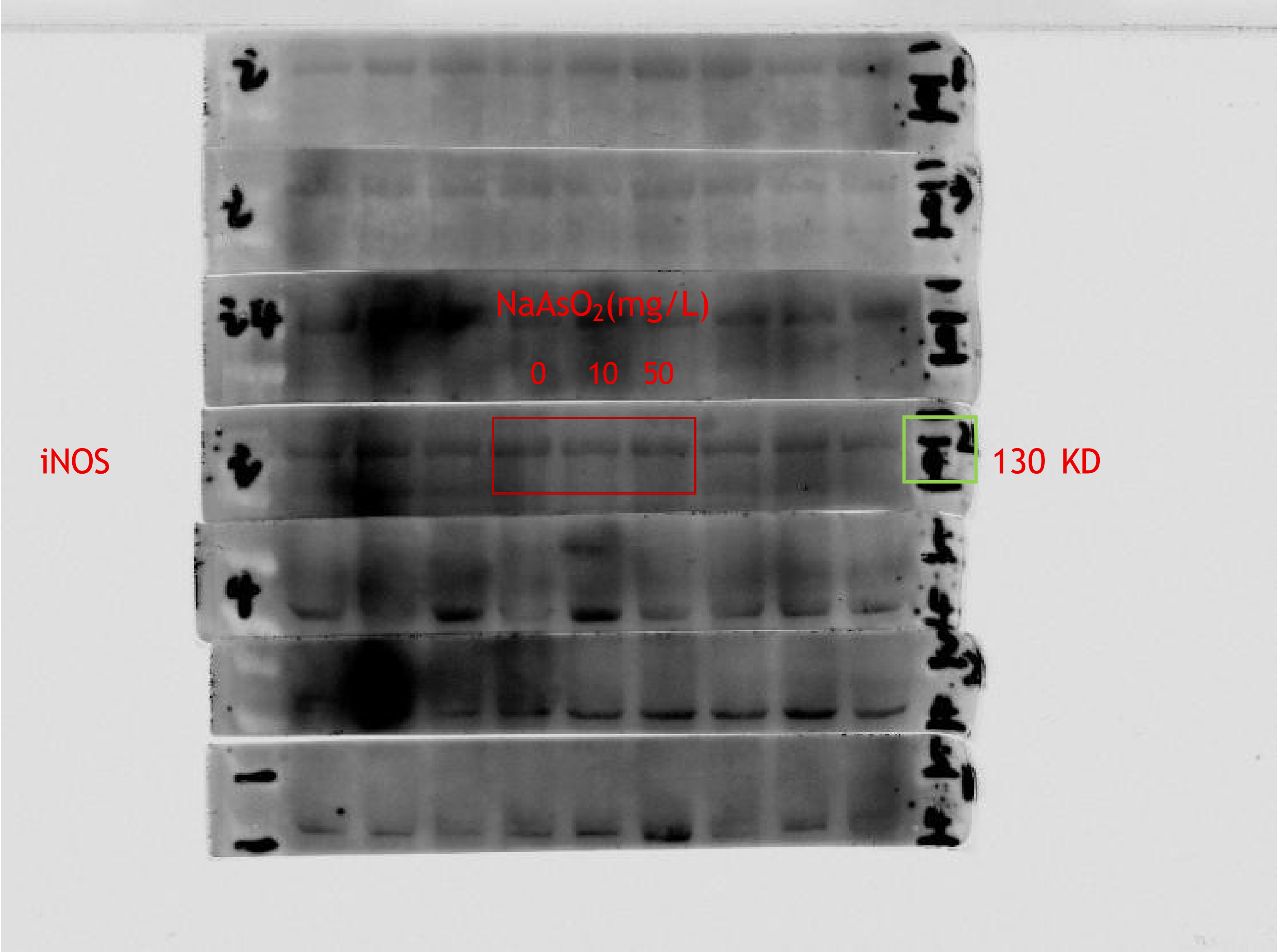

Fig1 e IL-1β

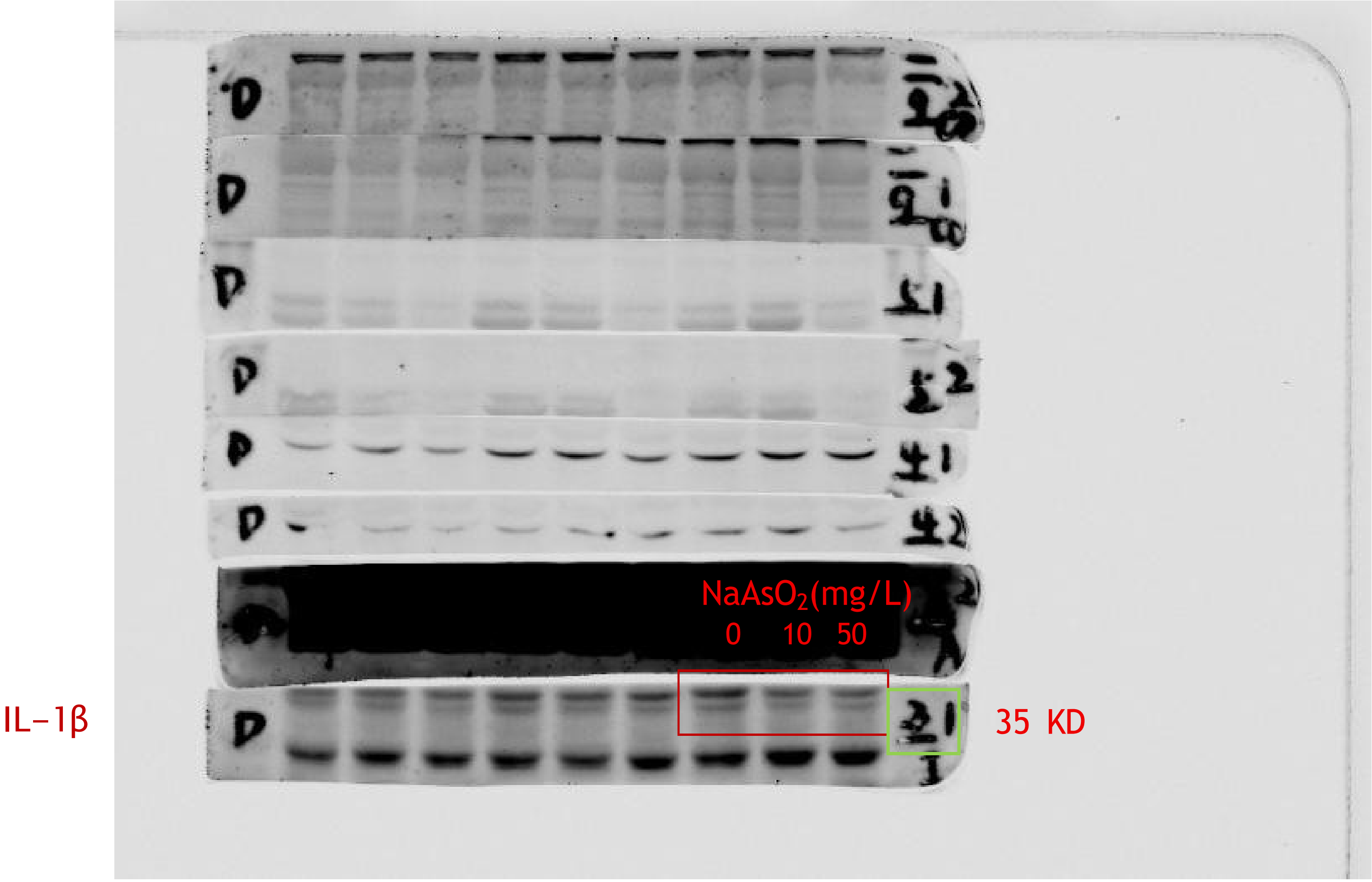

Fig1 e

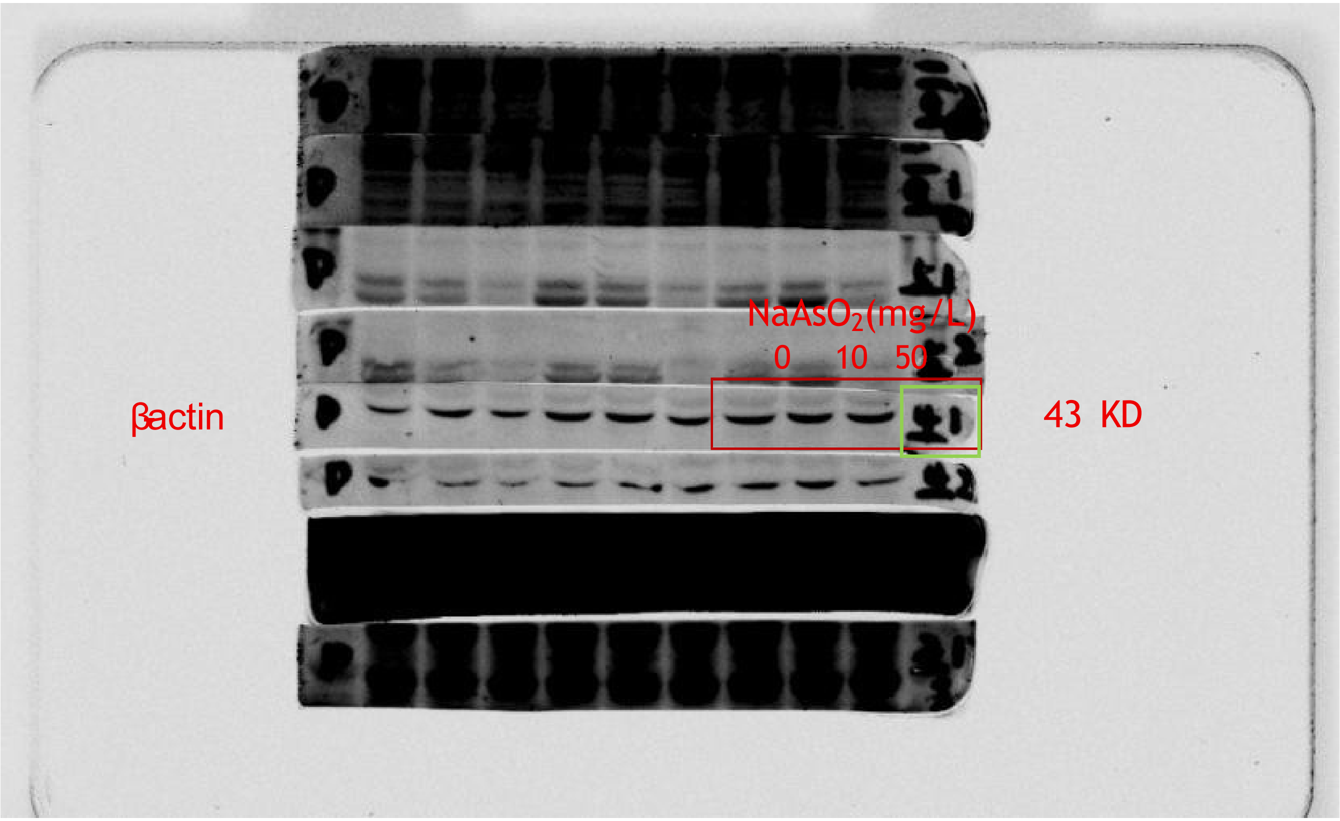

Fig3b

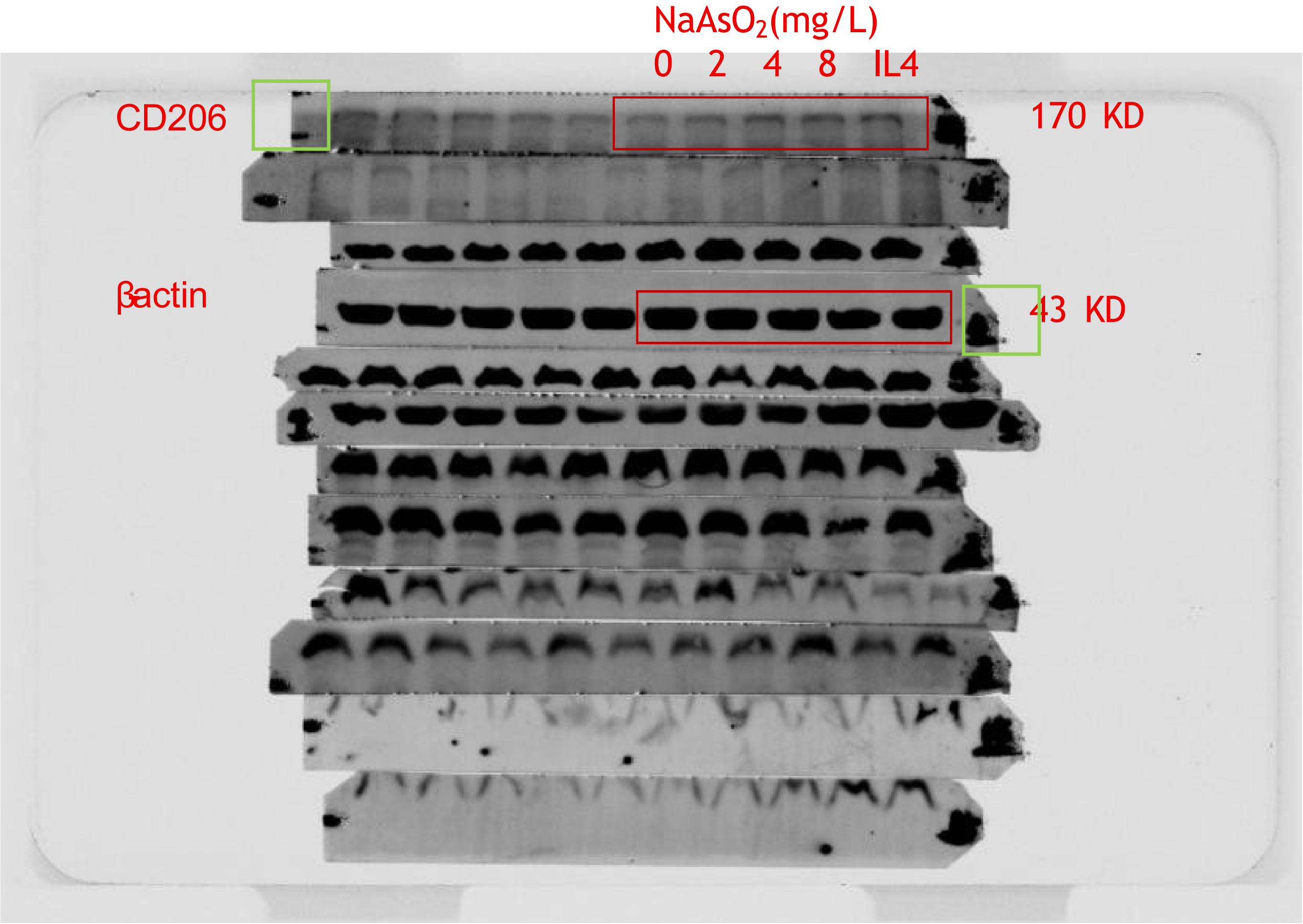

Fig3c

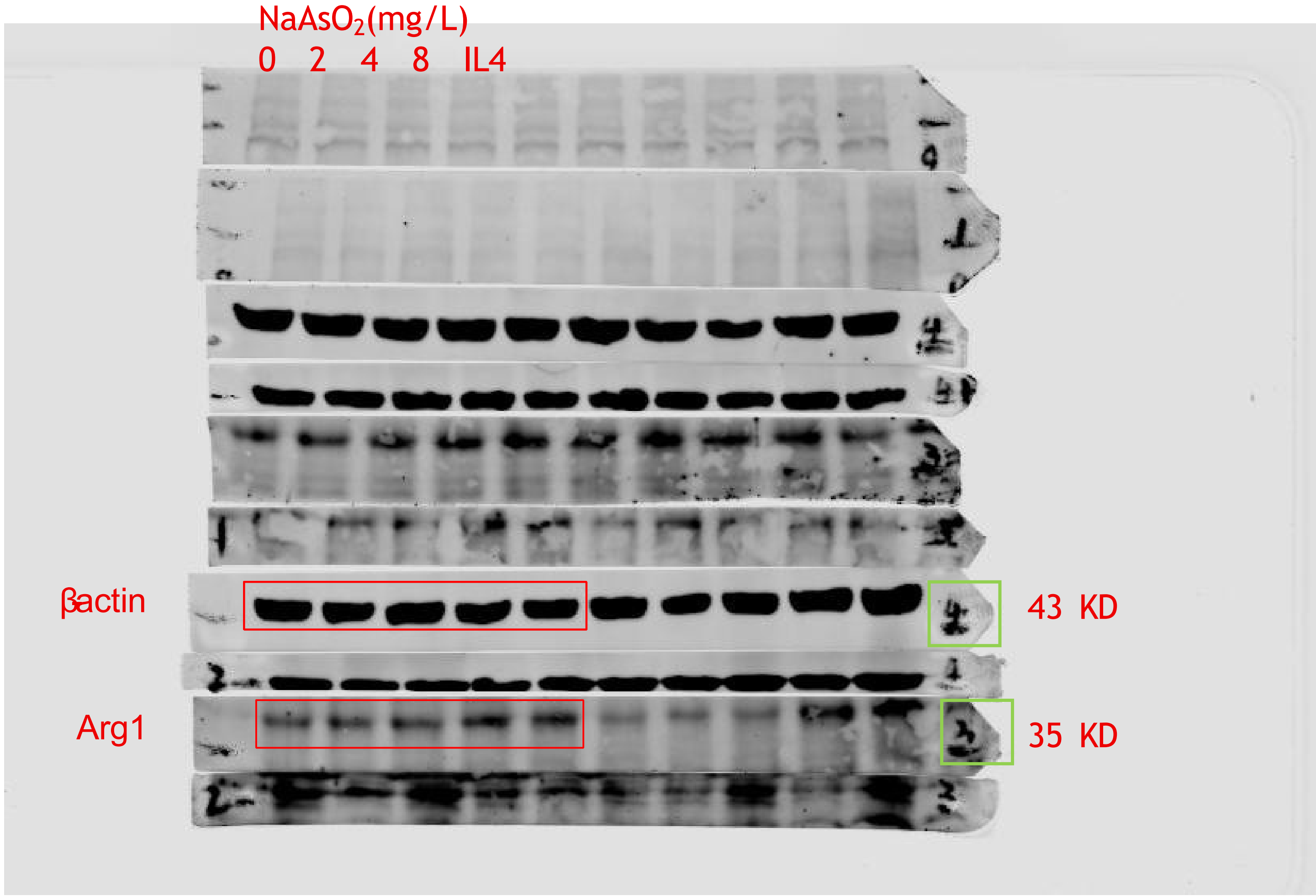

Fig3d

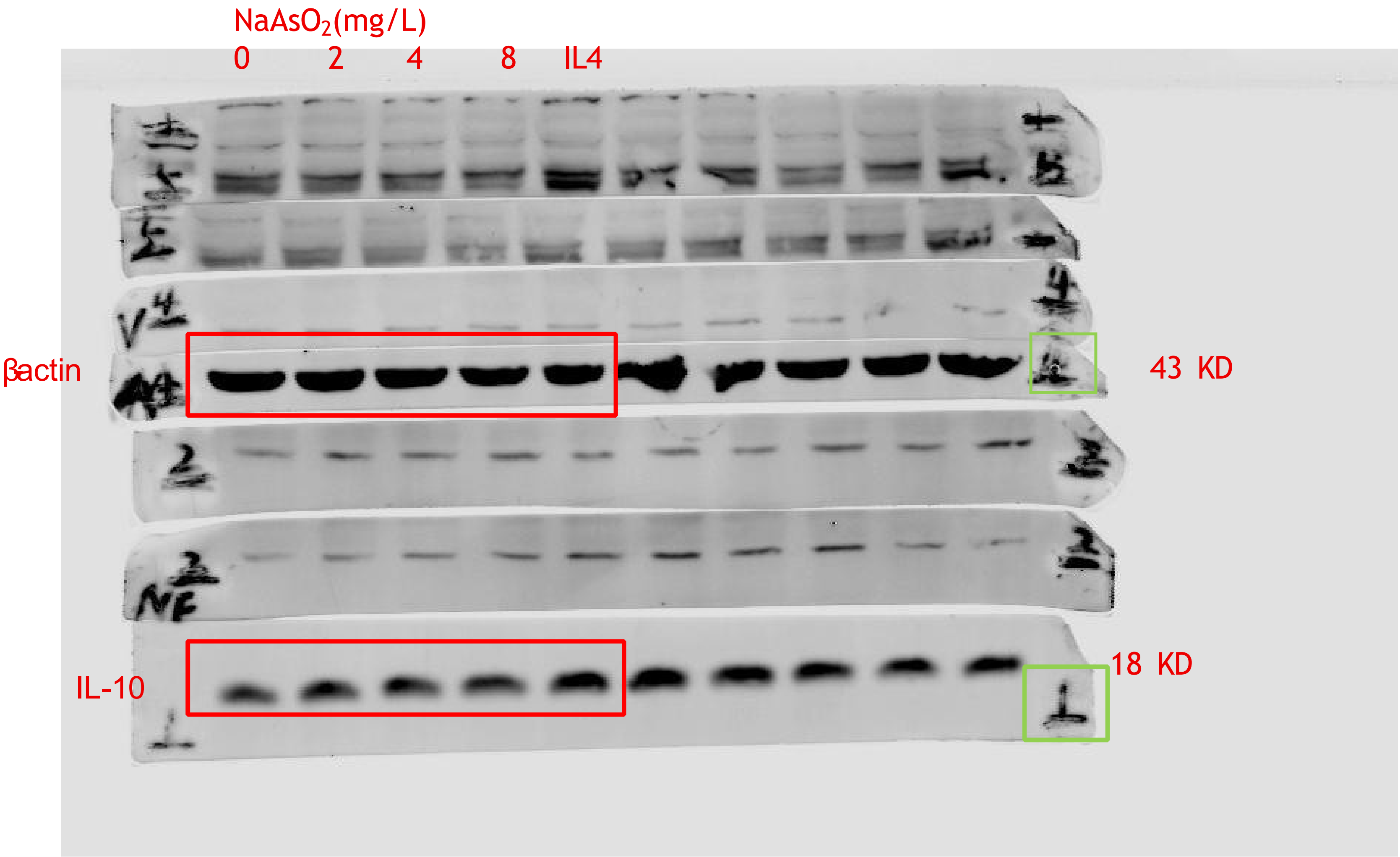

Fig3e

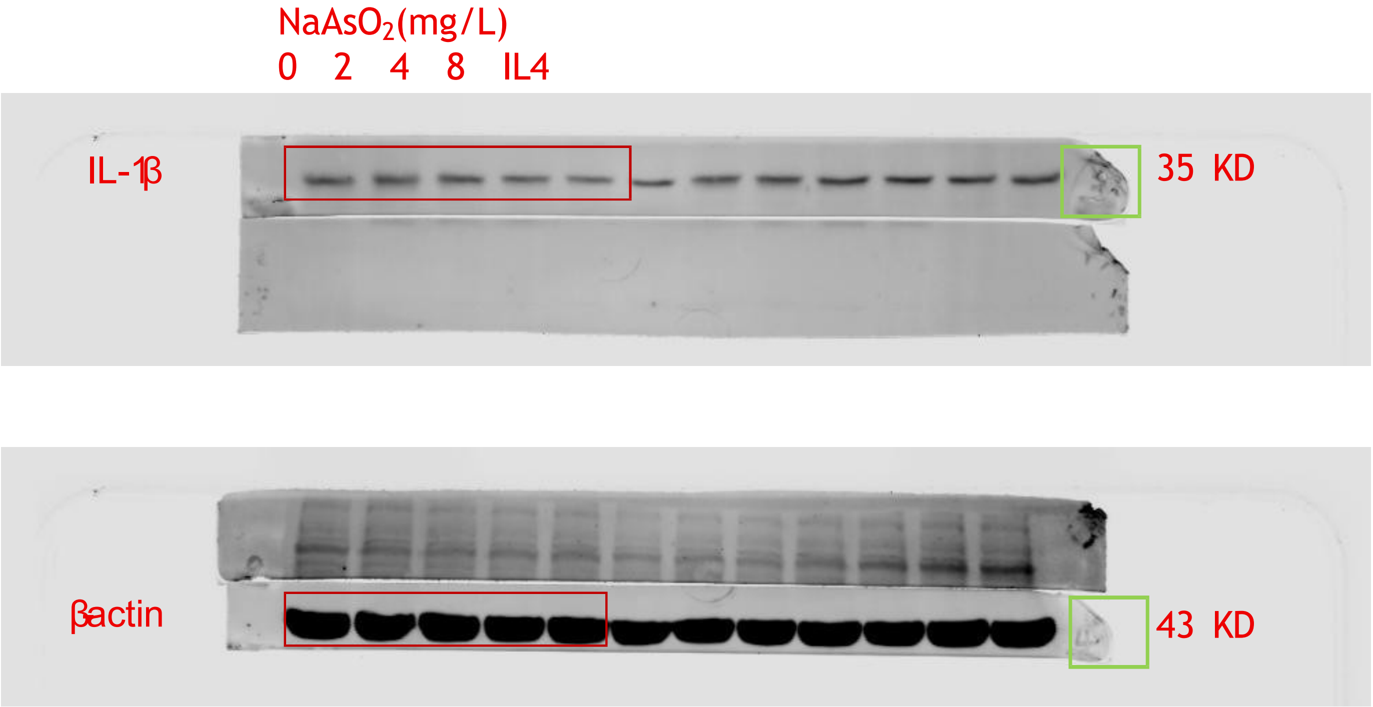

Fig3f TNFa

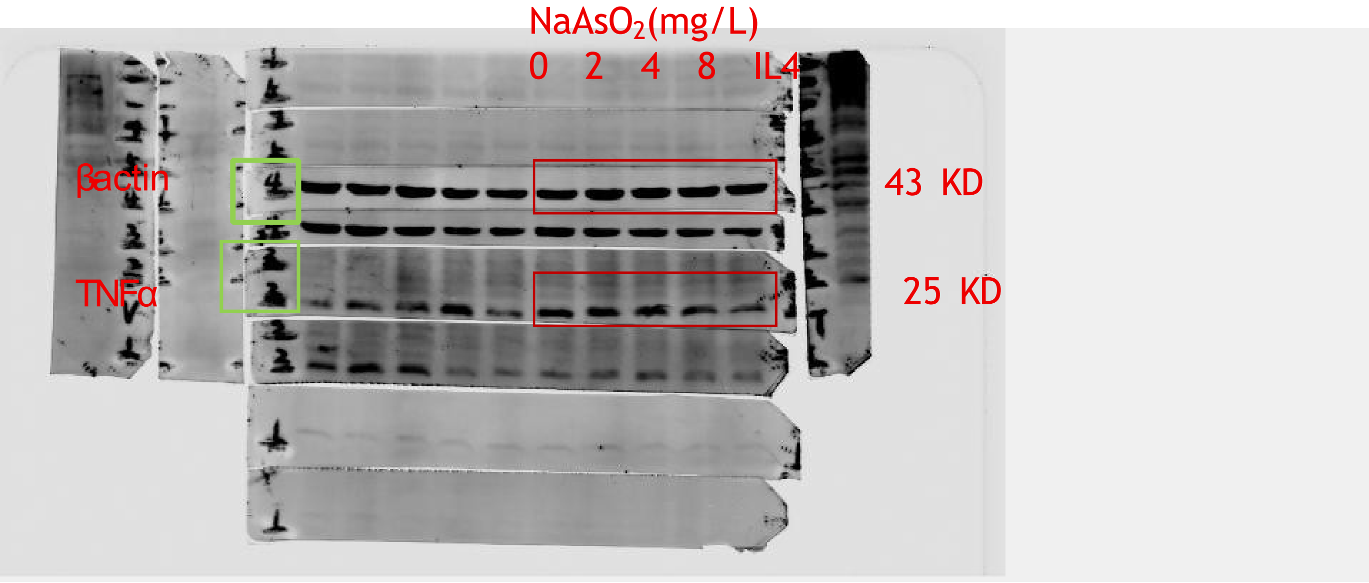

Fig3g iNOS

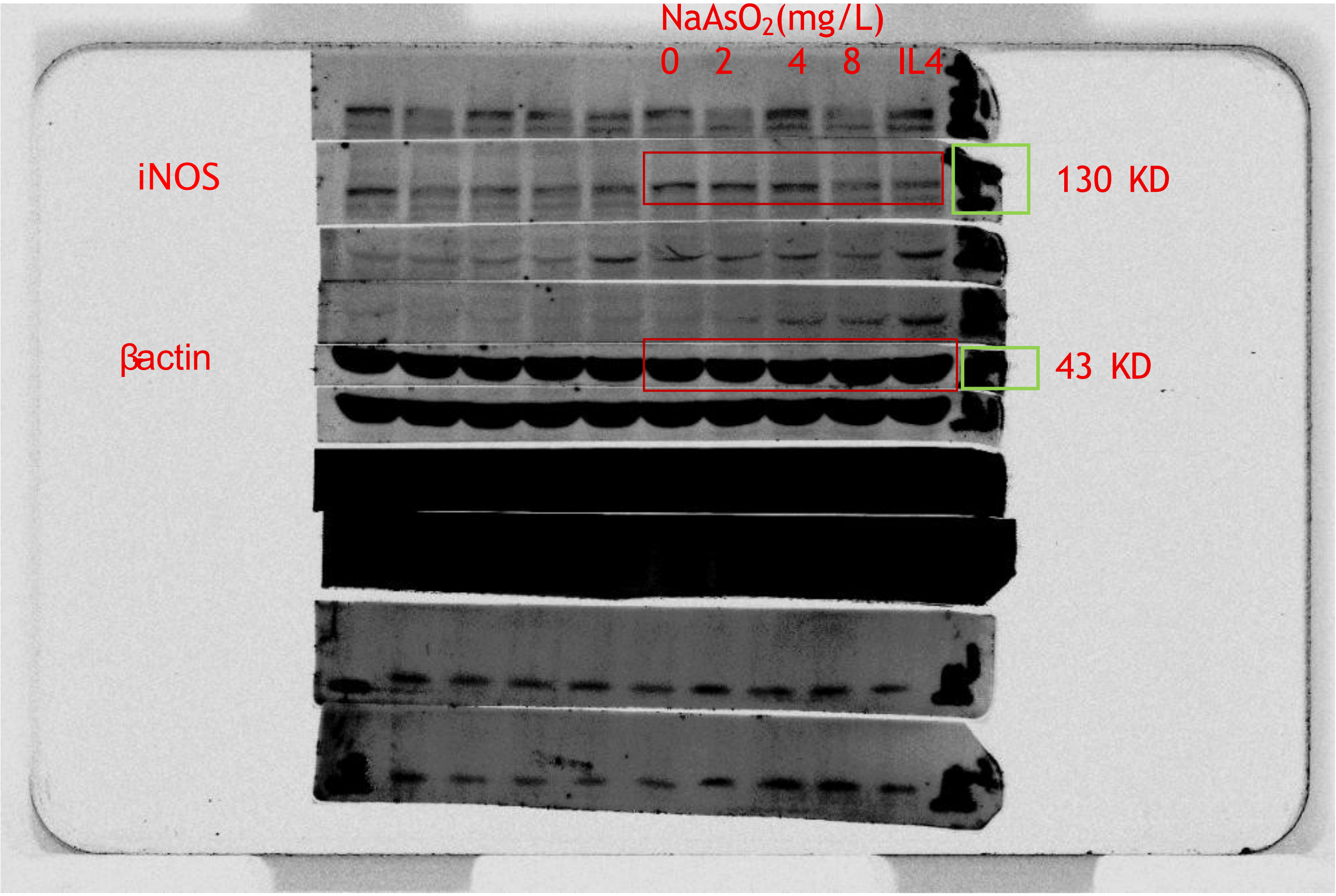

Fig3h VEGF

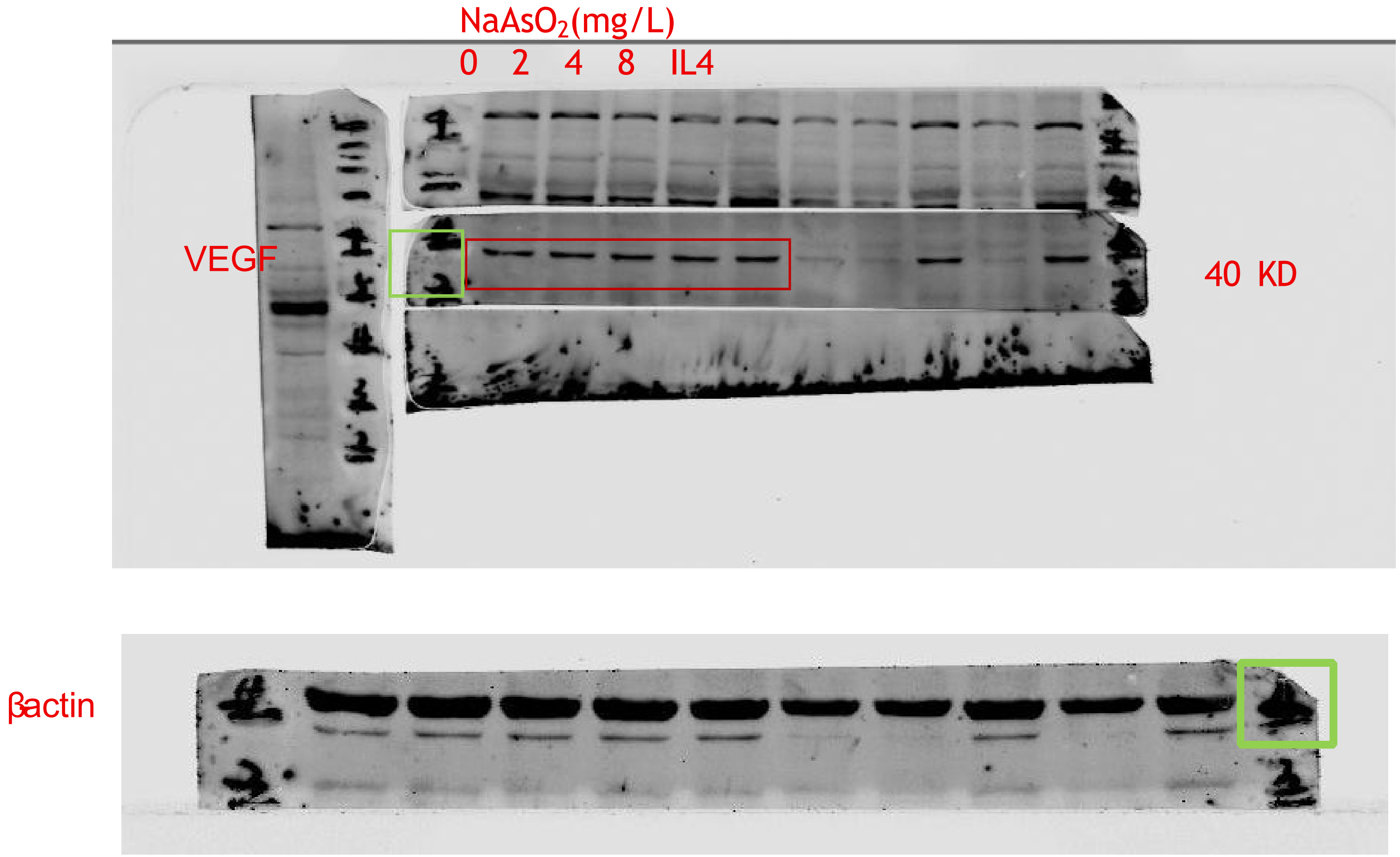

Fig6a IRF4

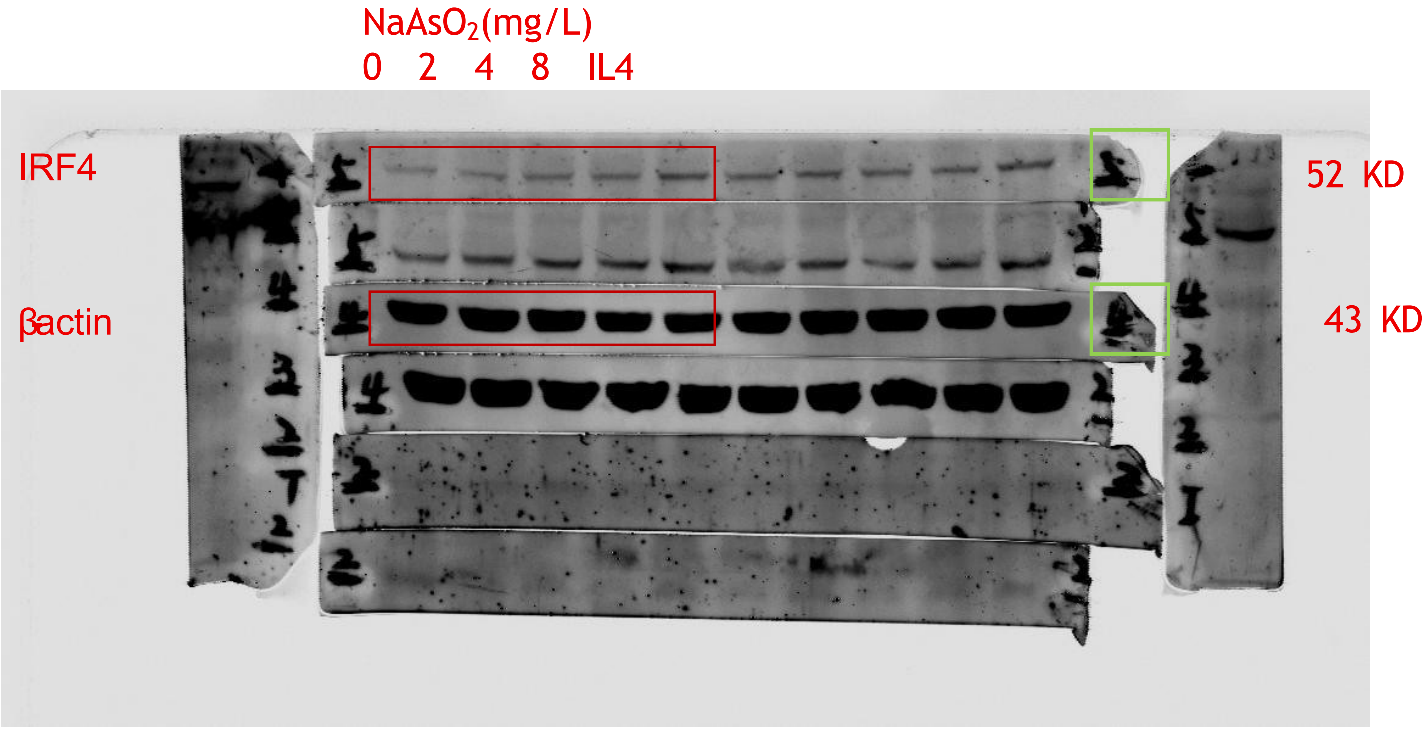

Fig6c IRF4

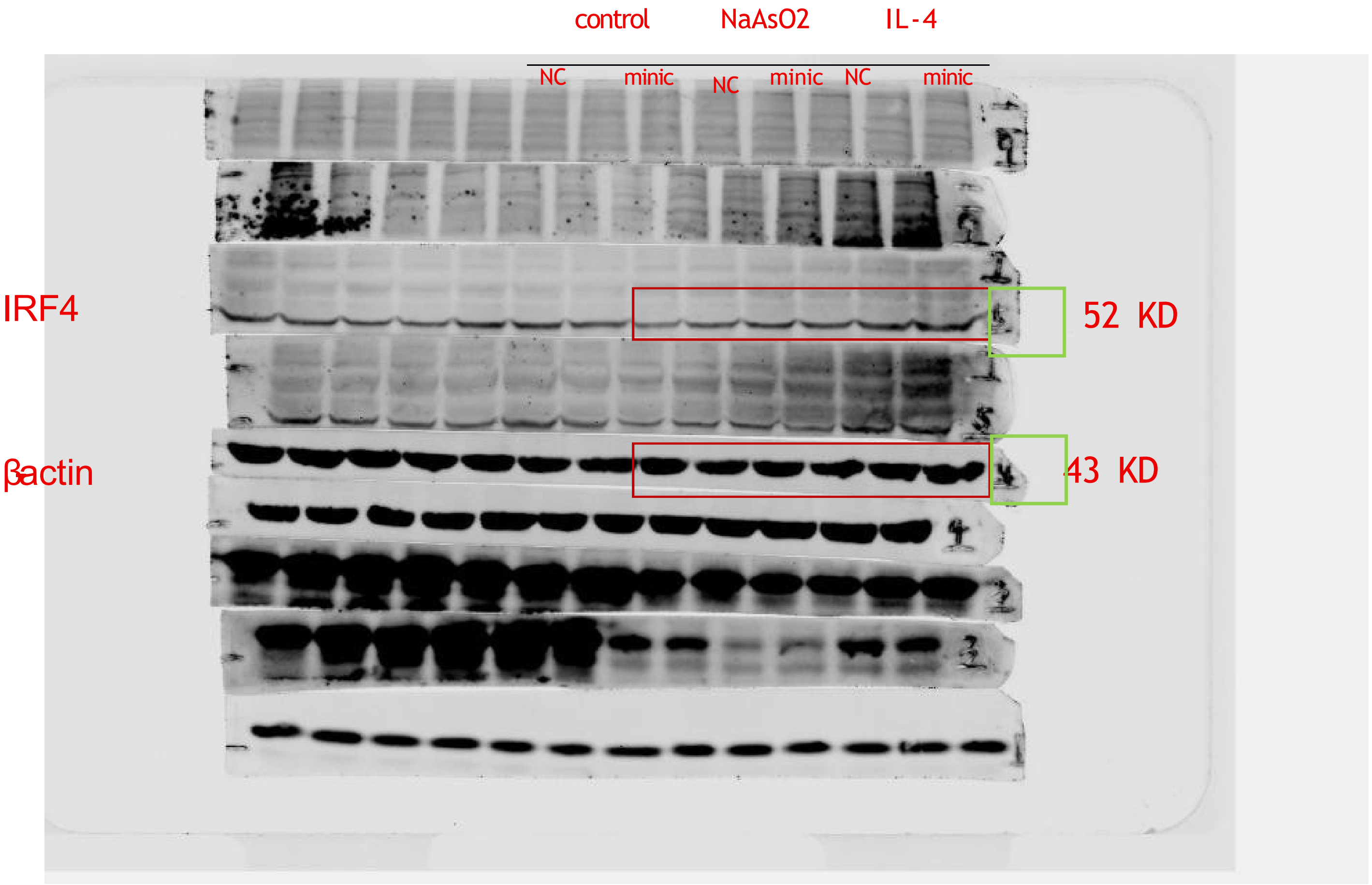
control
NaAsO2
IL-4
NC
minic
NC
minic
NC
minic
IRF4
52 KD
βactin
43 KD

Fig7a CD206

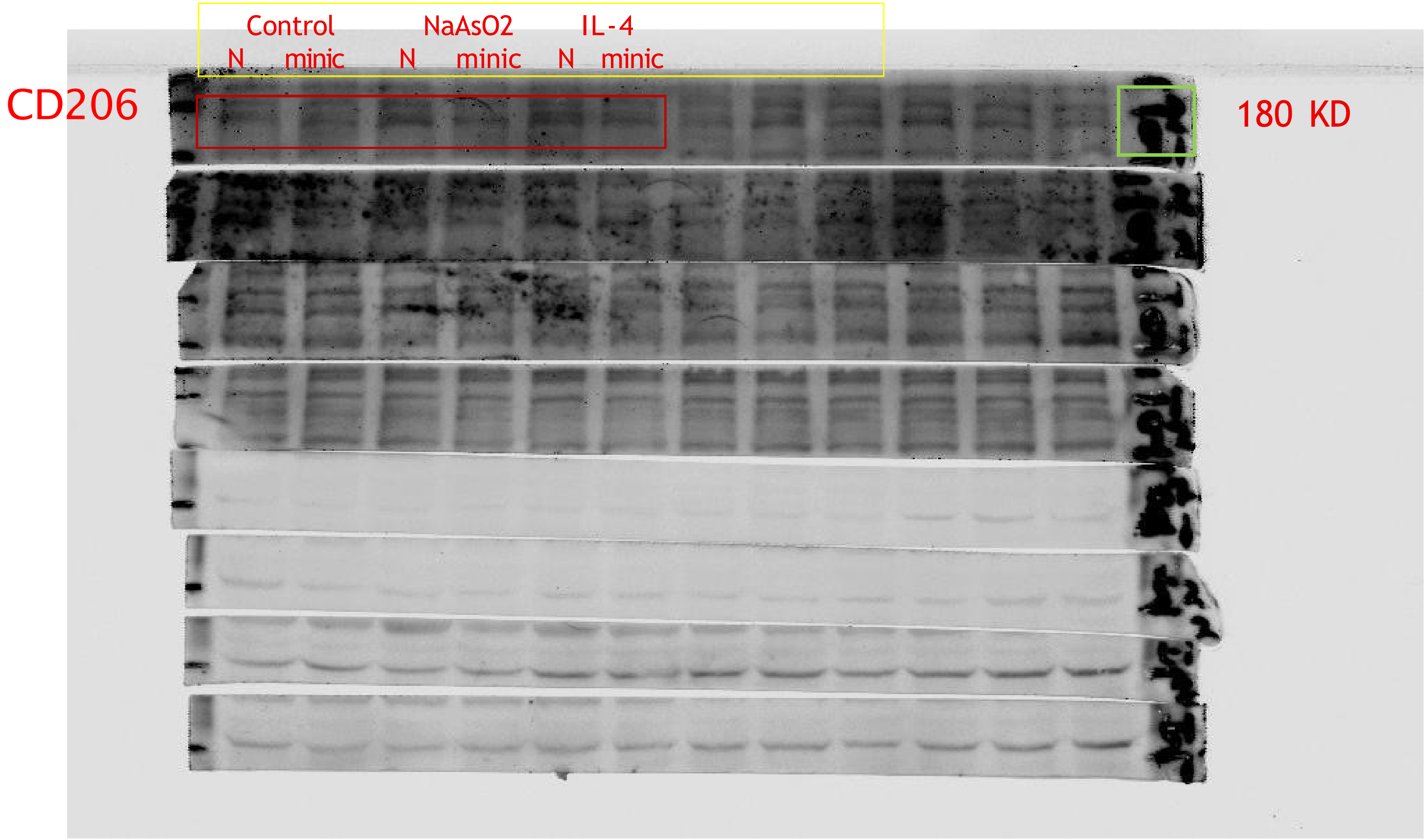

Fig 7a CD206 β-actin

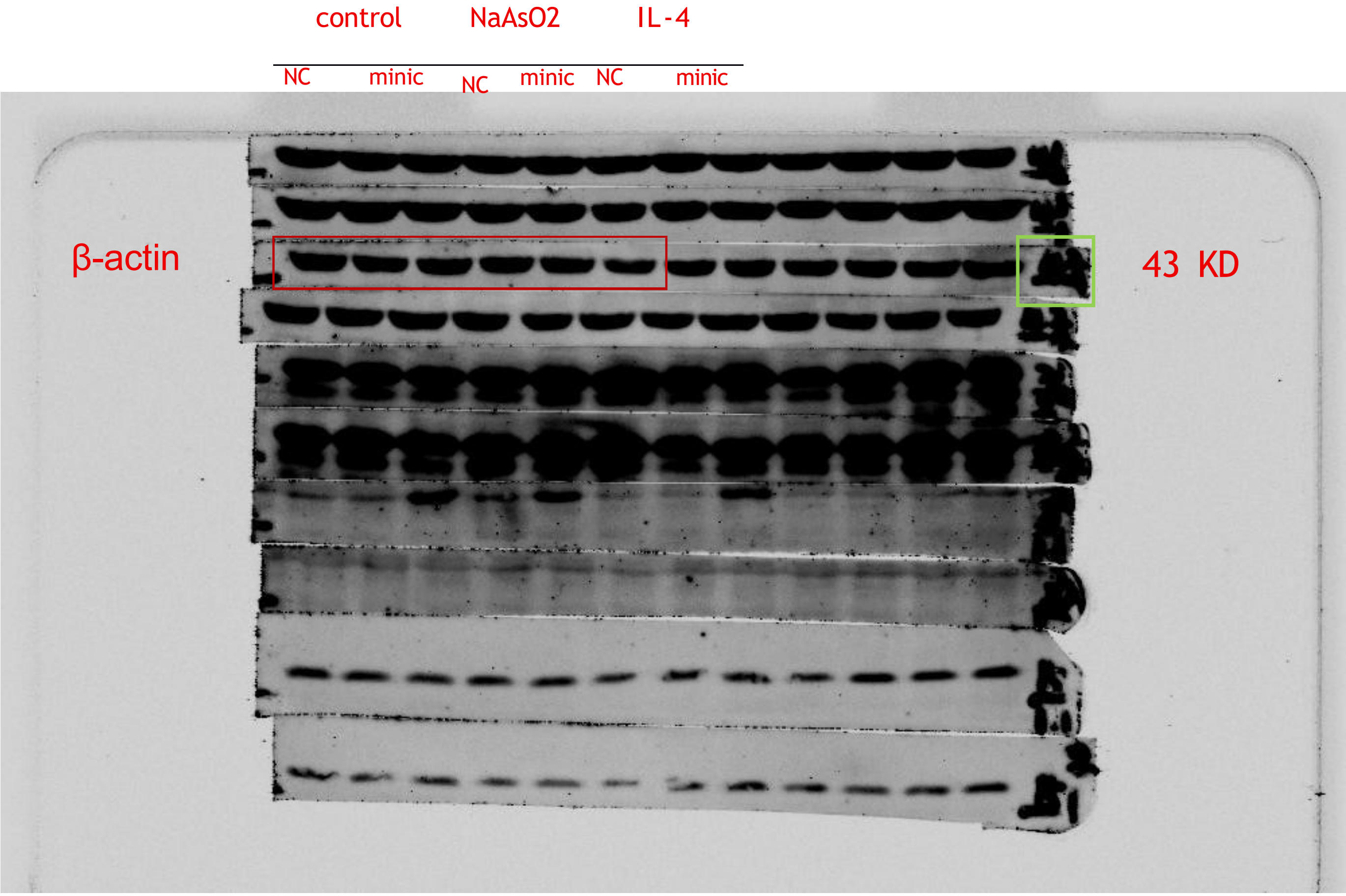

Fig7b Arg1

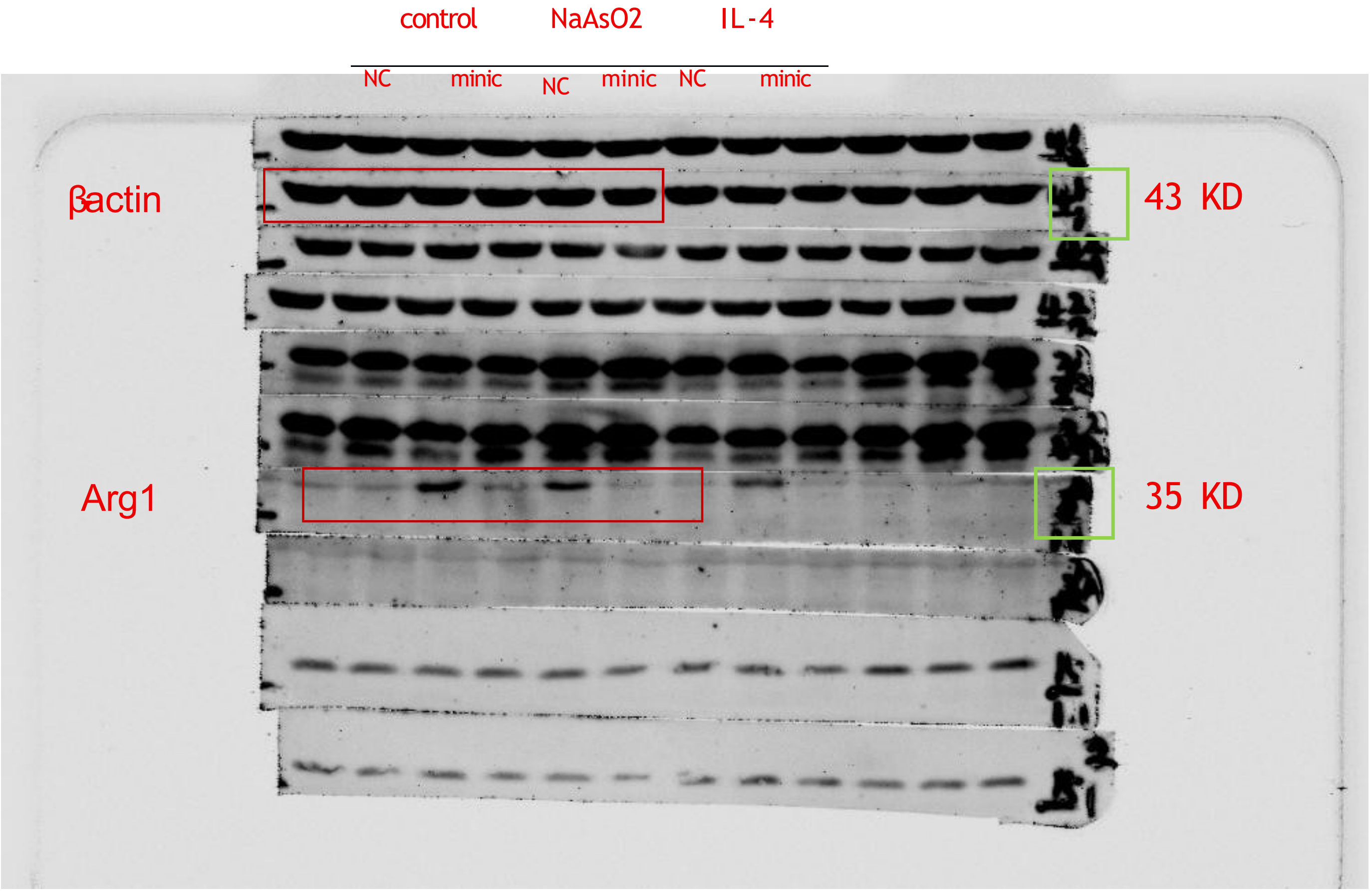
control
NaAsO2
IL-4
NC
minic
NC
minic
NC
minic
βactin
43 KD
Arg1
35 KD

Fig7c IL10

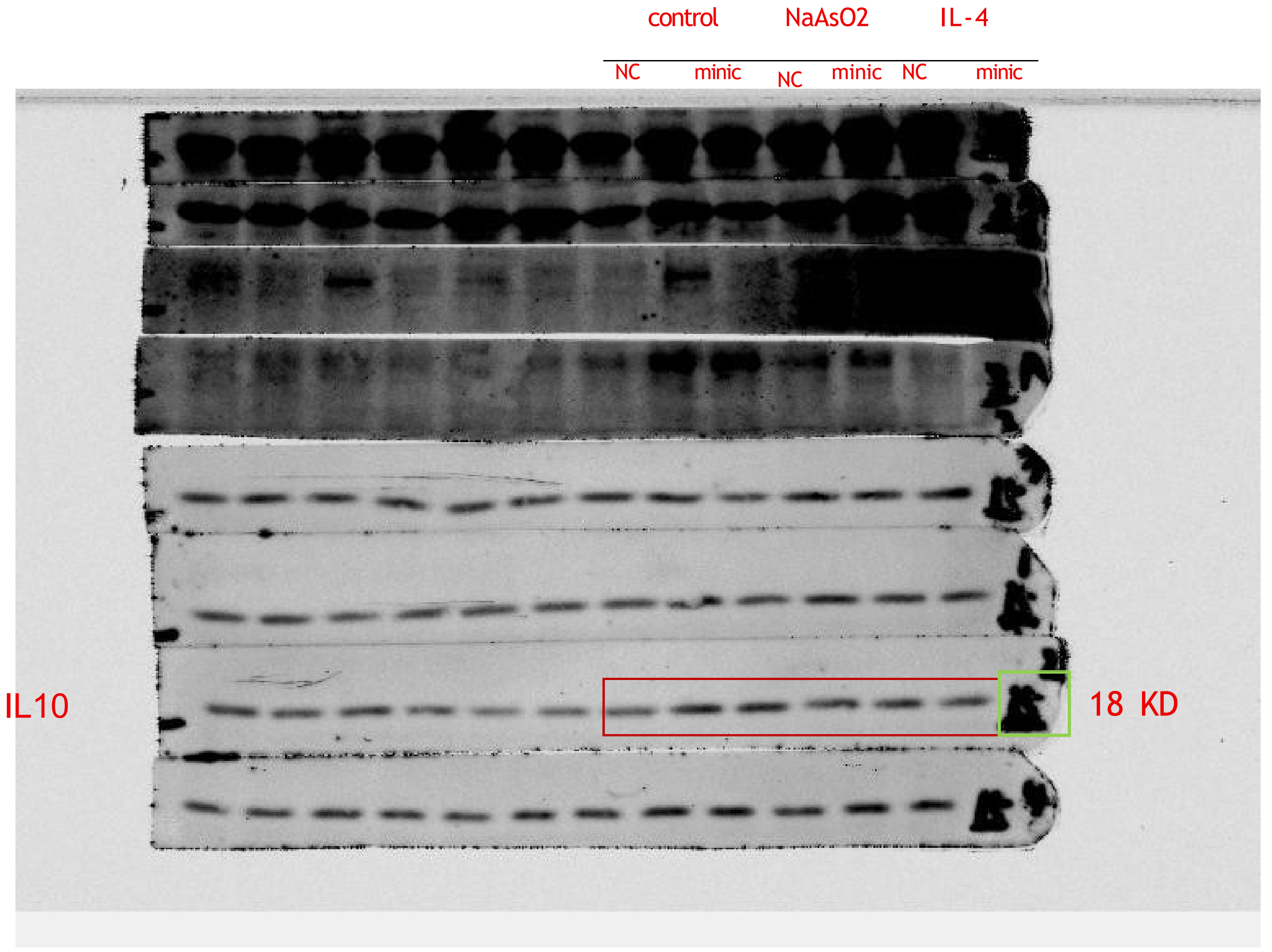

Fig7c IL10

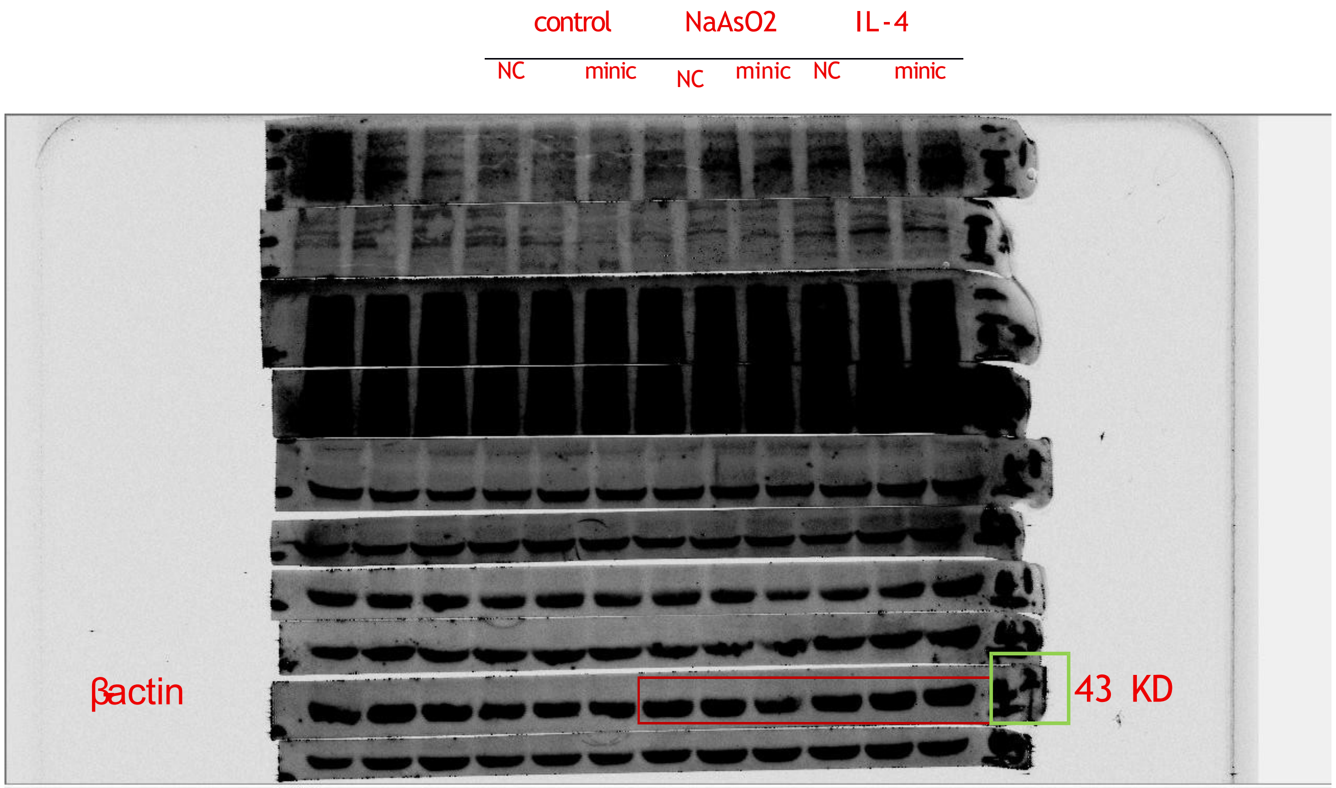

Fig8a iNOS

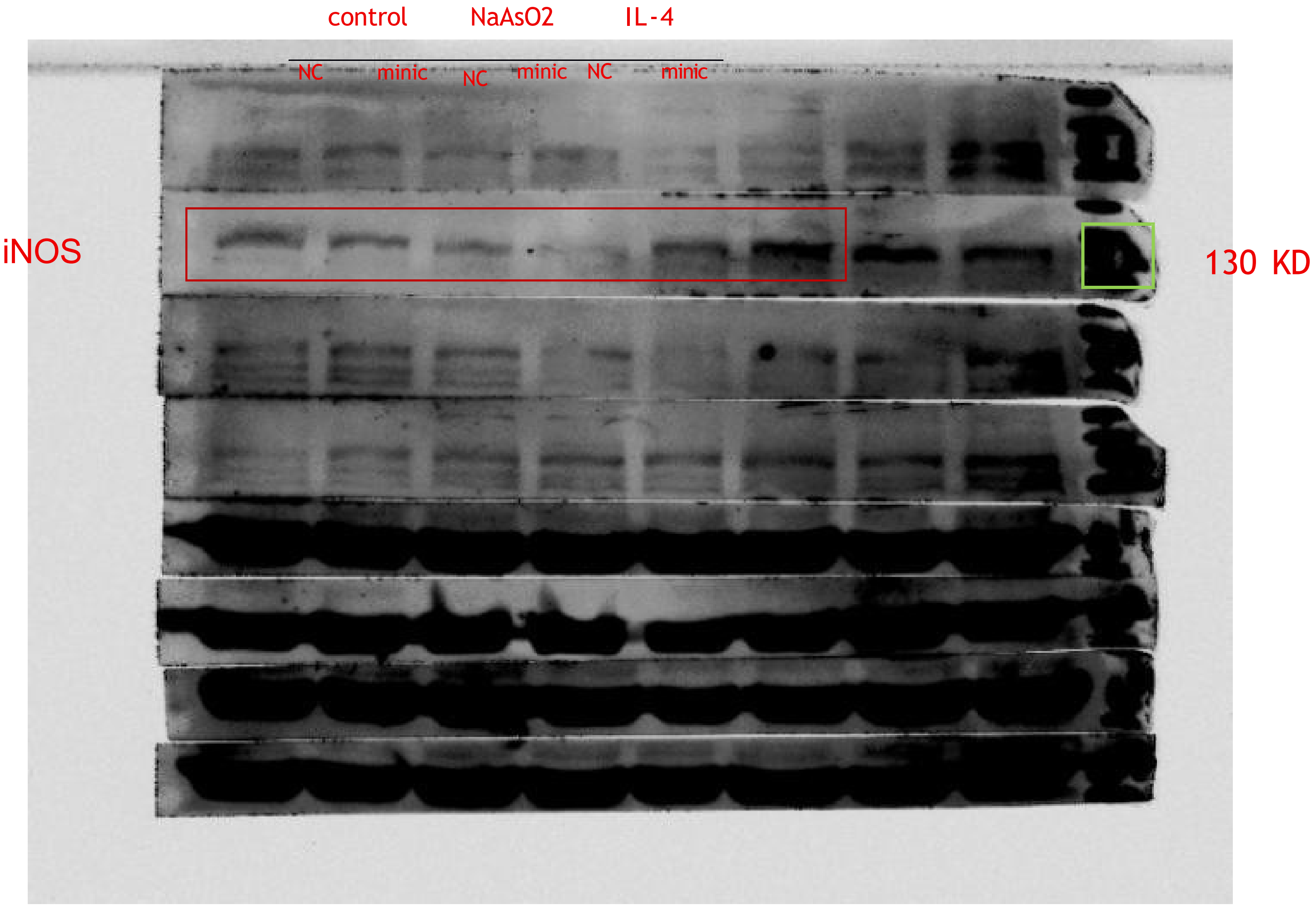

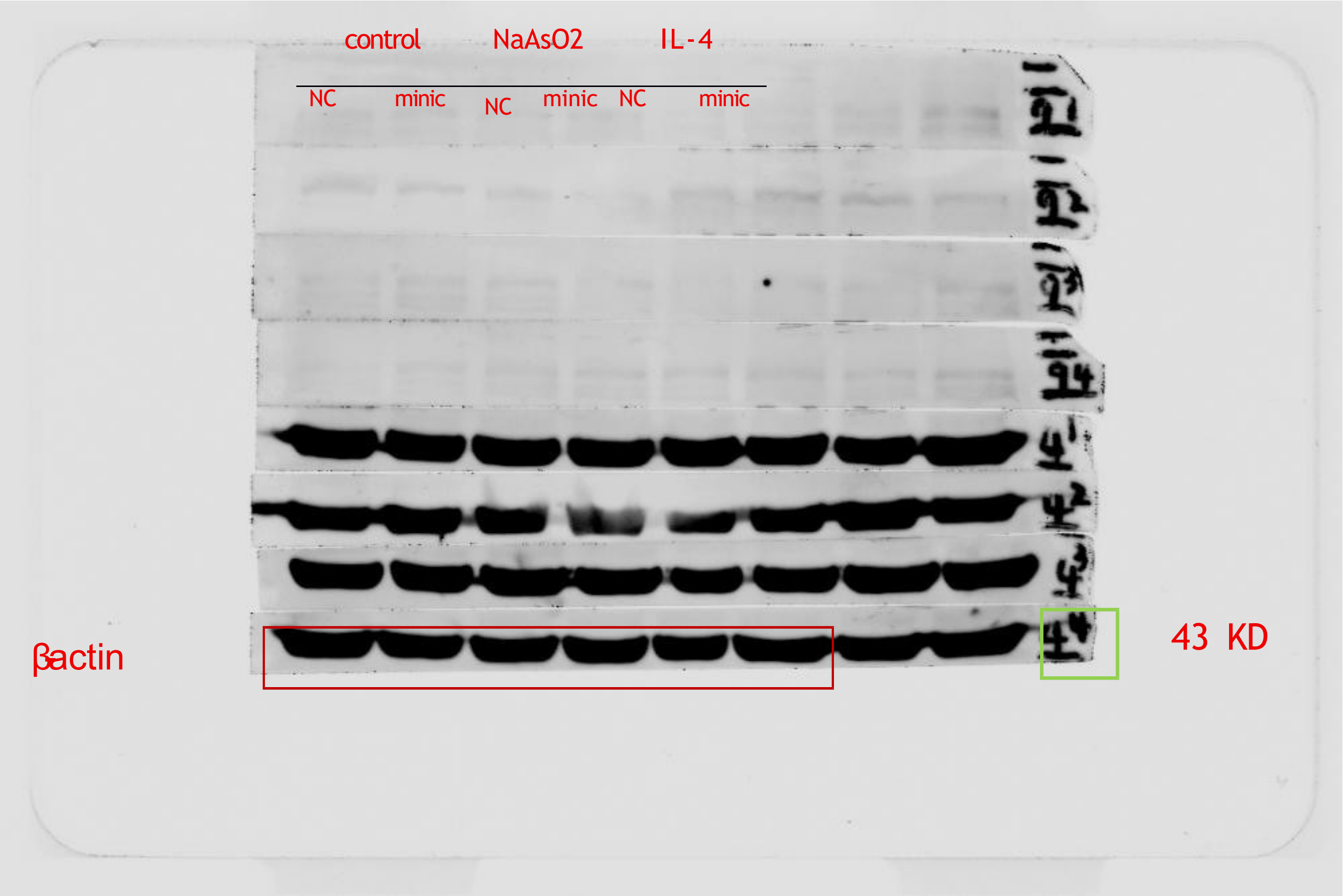

control
NaAsO2
IL-4
NC
minic
NC
minic
NC
minic
βactin
43 KD

Fig8b IL1β

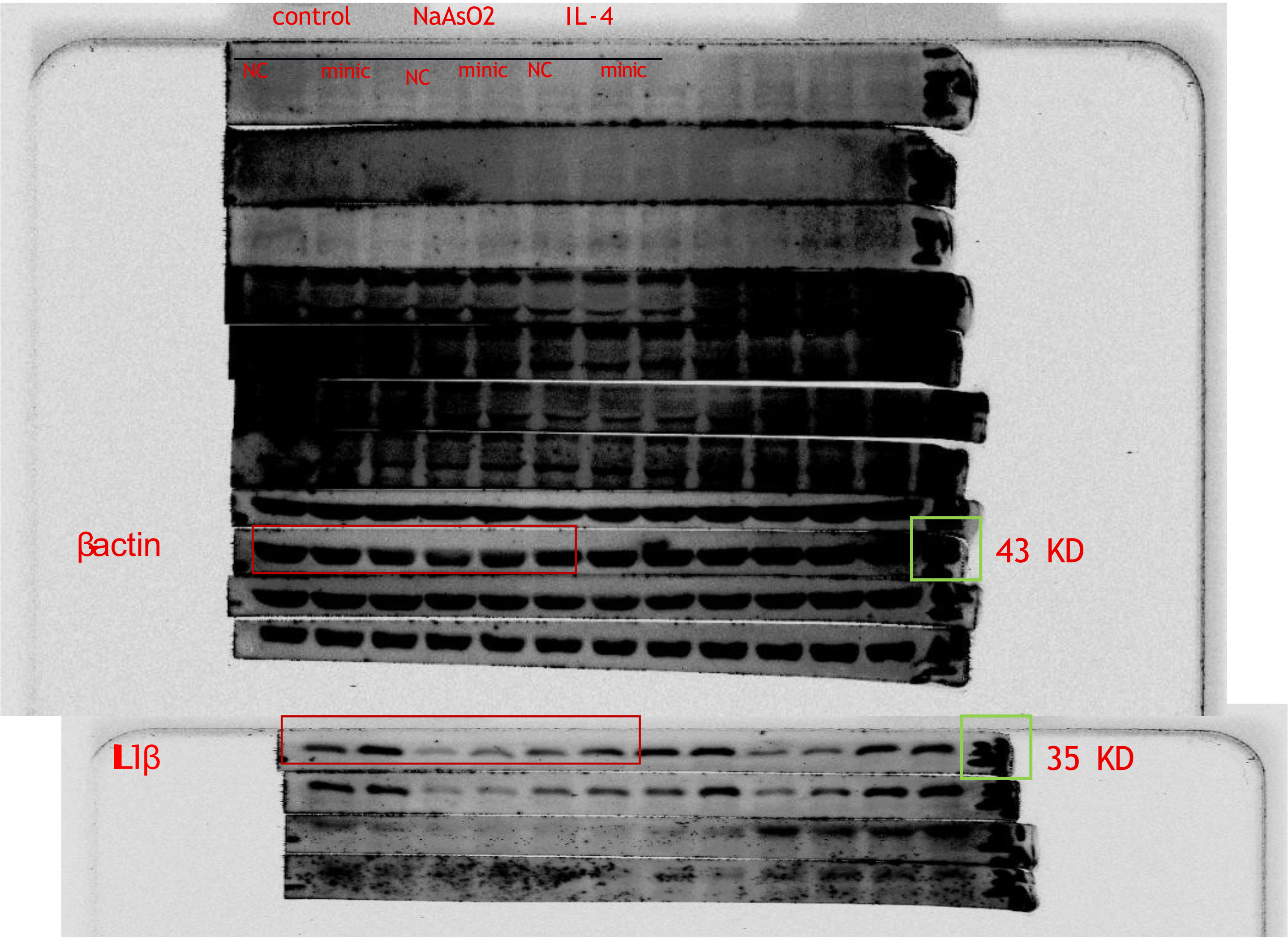
control
NaAsO2
IL-4
NC
minic
NC
minic
NC
minic
βactin
43 KD
IL1β
35 KD

Fig8c TNFα

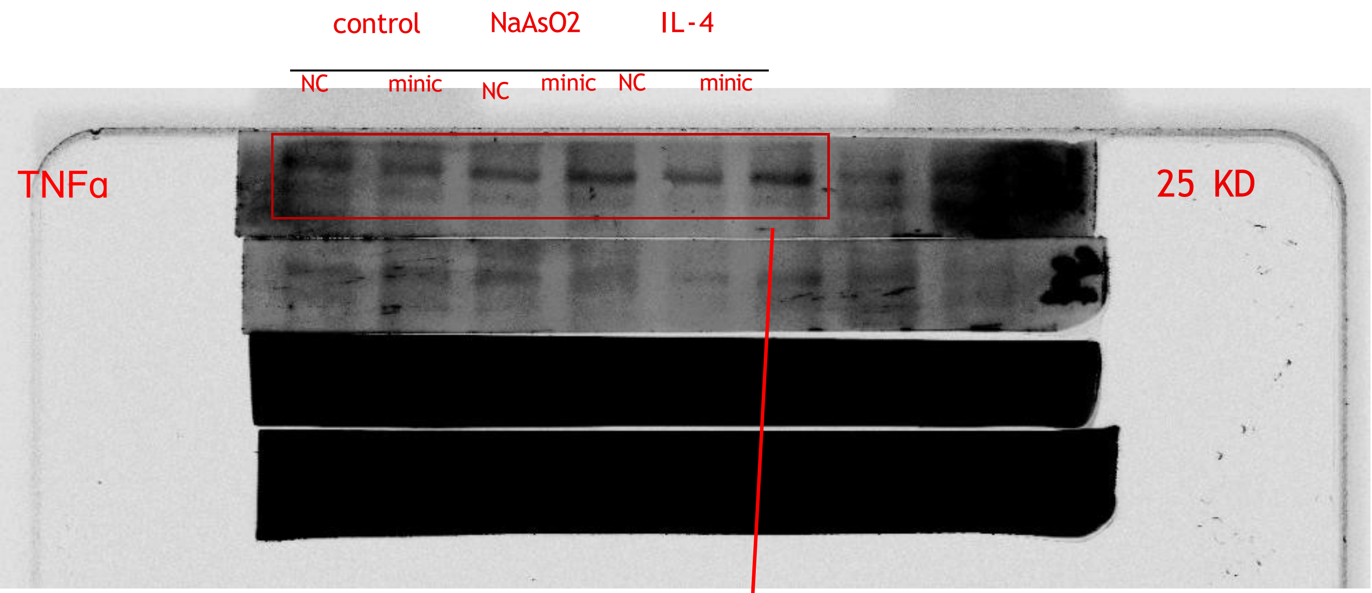

control
NaAsO2
IL-4
NC
minic
NC
minic
NC
minic
TNFα
25 KD

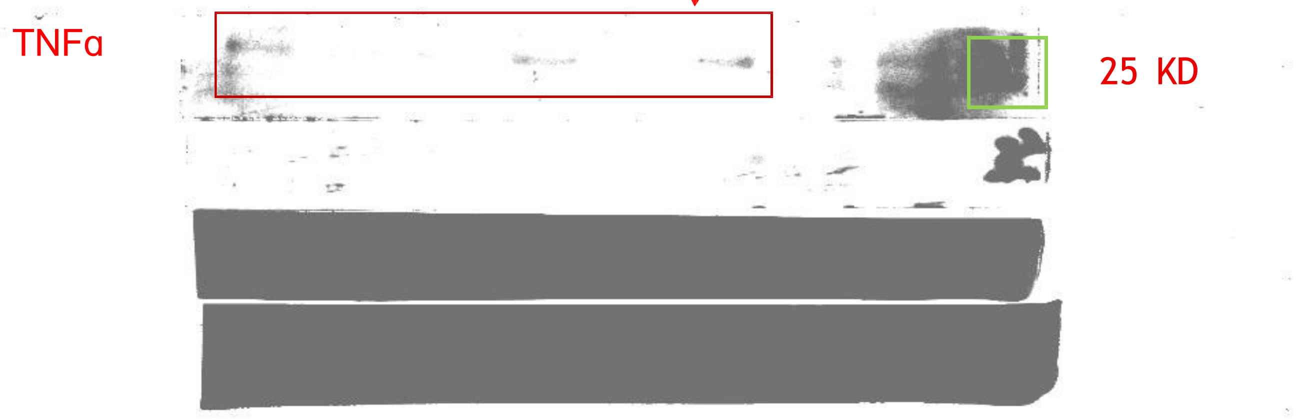

TNFα
25 KD

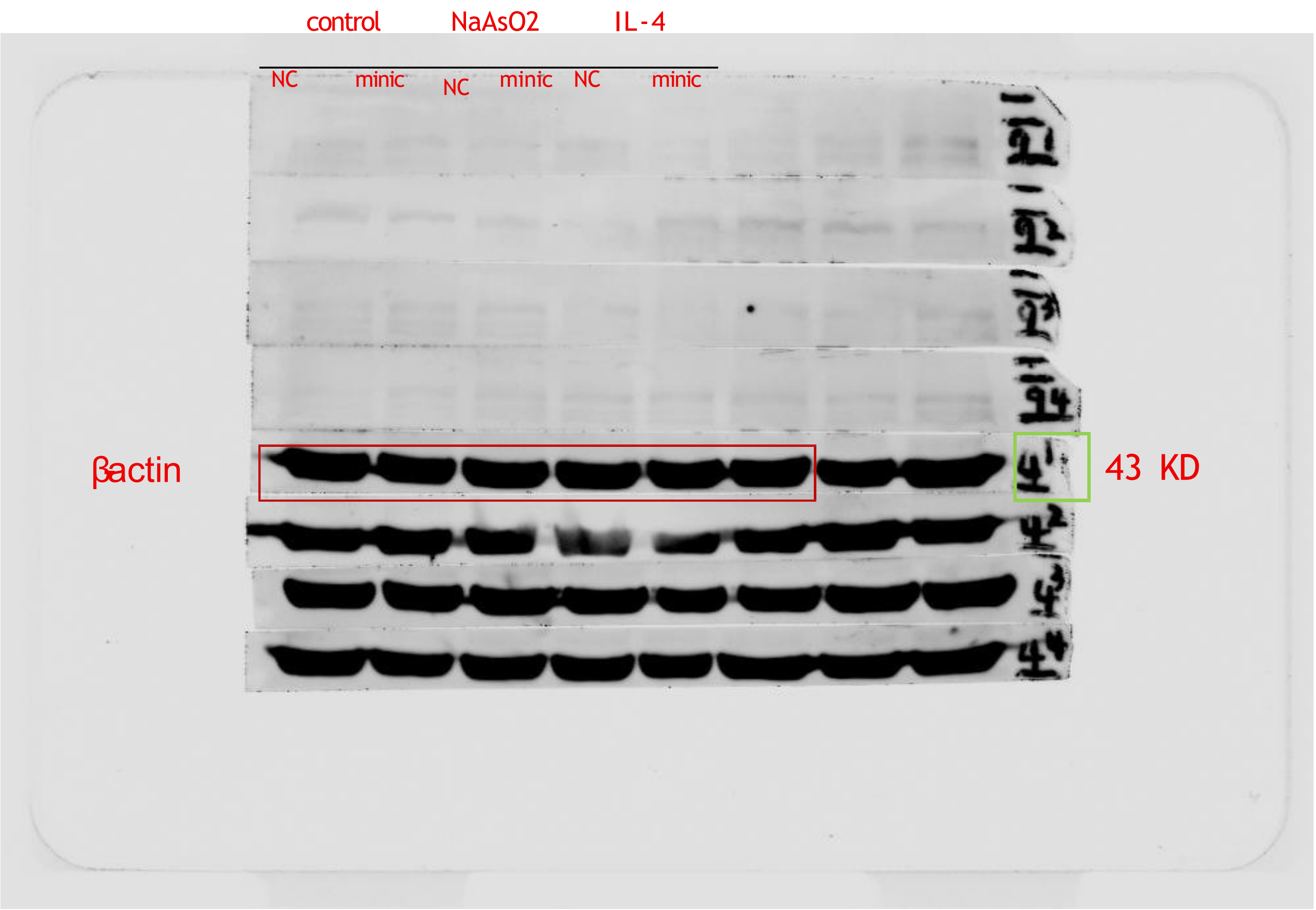
control
NaAsO2
IL-4
NC
minic
NC
minic
NC
minic
βactin
43 KD
