# Supplementary material for: The miR-125a-5p/IRF4 Axis Mediates Sodium Arsenite-Induced M2 Macrophage Polarization
Source: Biomolecules. 2025 Nov 20;15(11):1630. doi: 10.3390/biom15111630 (PMC12649919; doi:10.3390/biom15111630)
Supplement: Supplementary file 1 [file biomolecules-15-01630-s001.zip › biomolecules-3939521-supplementary.pdf]

Supplementary Material

Morphological characteristics of THP-1 cells after differentiation

THP-1 monocytes showed macrophage-like morphological differentiation after 48h stimulation with PMA. Compared to untreated controls, PMA-treated cells transitioned from a suspension state to adherent growth, accompanied by pseudopodia extension, cessation of mitotic activity, and cytoplasmic expansion (Fig S a and S b). The expression level of CD11b, a macrophage surface marker, was detected by flow cytometry. In the absence of any stimulation, THP-1 cultured cells with 30 % CD11b. After treatment with different concentrations of PMA, we observed a significant increase in the number of CD11b cells (Fig S c-e). The results showed that M0 macrophages were successfully obtained from human mononuclear cell line THP-1. The induction effect of 80 nM PMA and 160 nM PMA was compared (Fig S f), and there was no statistical difference between the two dose groups. 80 nM PMA was selected to induce THP-1 cells.

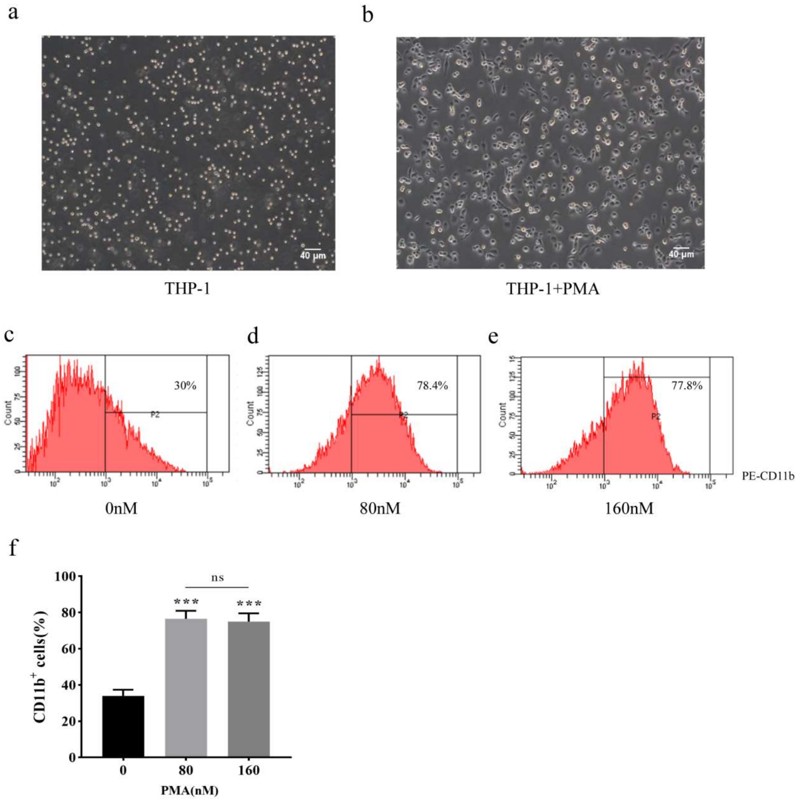

Figure S1. Morphological changes of THP-1 cells treated with PMA and expression of macrophage marker CD11b. (a -b) Morphological changes of THP-1 cells treated with PMA (4 x). (c, d and e) Flow cytometry were used to detect CD11b expression in macrophages induced by different concentrations of PMA. (f) Quantified statistics of macrophage CD11b induced by different concentrations of PMA. Compared with control group. Data were presented as mean ± SD. * P< 0.05; ** P< 0.01; *** P<0.001.
